# Catalysts, Mechanisms, and Challenges in Methane (CH_4_) Decomposition

**DOI:** 10.3390/ma19163438

**Published:** 2026-08-13

**Authors:** Magdalena Jabłońska, Marek Rotko

**Affiliations:** 1 Department of Inorganic and Analytical Chemistry, Faculty of Chemistry, University of Łódź, Tamka 12, 91-403 Łódź, Poland; 2 Department of Chemical Technology, Institute of Chemical Sciences, Faculty of Chemistry, Maria Curie-Skłodowska University, Maria Curie-Skłodowska Sq. 3, 20-031 Lublin, Poland; marek.rotko@umcs.pl

**Keywords:** CH_4_ decomposition, hydrogen production, transition metal catalysts, catalyst deactivation and regeneration

## Abstract

A key challenge facing modern society, fueled by the relentless growth in energy and food requirements, is meeting rising energy needs without exacerbating greenhouse gas emissions. Nevertheless, fossil fuel combustion remains the primary contributor to human-induced pollution. As environmental concerns intensify and fossil resources become increasingly scarce, there is a growing push within the research community to identify alternative energy carriers and to advance more sustainable, low-impact technologies. Thus, this review focuses on catalytic CH_4_ decomposition (CDM) for hydrogen production over Ni-, Fe, and Co-metal-based catalysts. Fe-based catalysts have received considerable attention for CDM due to their low cost and environmental sustainability. Furthermore, a discussion of deactivation and regeneration, along with the identified reaction mechanisms of CH_4_ decomposition over these catalysts, is presented.

## 1. Introduction

Hydrogen is widely recognized as a promising energy carrier. When used in combustion, it has only one by-product: water [1,2], making it an attractive option for reducing emissions. Currently, hydrogen is widely utilized as both a chemical feedstock and an energy carrier in the chemical and petroleum industries, where it is essential to produce key chemicals such as ammonia and methanol. Moreover, hydrogen holds significant potential across a range of future applications—most notably in transportation—where its use as a fuel is gaining momentum alongside the rapid development of fuel cell technologies [3,4]. With a gravimetric calorific value of 123 MJ kg^−1^, hydrogen exhibits the highest energy content among all fuels, approximately three times that of gasoline. Furthermore, hydrogen combustion does not generate environmentally harmful emissions, including CO, CO_2_, and fine particulate matter [5].

One of the principal advantages of hydrogen as an energy carrier is the diversity of its production pathways. Hydrogen can be produced from a wide variety of primary energy sources, including fossil fuels, renewable energy, and nuclear power. However, the envisioned hydrogen economy—where hydrogen serves as a major energy carrier produced predominantly from renewable resources—remains a long-term goal. At present, hydrogen production from renewable energy sources remains significantly more expensive than conventional production methods, and substantial cost reductions are required before it can become economically competitive at scale. Conventional industrial hydrogen production methods, such as steam methane reforming, partial oxidation, and autothermal reforming of natural gas, are associated with substantial emissions of carbon oxides, particularly CO_2_, into the atmosphere [6,7,8]. Among the alternative hydrogen production routes, the thermal and catalytic decomposition of methane under oxygen-free conditions has attracted considerable research interest as a potential substitute for conventional processes. CH_4_, the major constituent of natural gas, is recognized as a powerful greenhouse gas, exhibiting a global warming potential more than 20 times higher than that of CO_2_. At the same time, methane represents an abundant and relatively inexpensive carbon resource. Methane serves as an important feedstock for a variety of industrial chemical processes, such as steam reforming, ammonia synthesis, and methanol production. However, these processes release large amounts of greenhouse gases, particularly CO_2_, leading to serious environmental pollution issues.

Catalytic methane decomposition is a promising hydrogen production technology capable of generating high-purity hydrogen with CO_2_ emissions as low as 4.37 t CO_2_ per t of H_2_ [9,10]. Consequently, CDM has attracted increasing attention as a promising route for hydrogen production without direct CO_2_ emissions. In this process, methane is decomposed into hydrogen and solid carbon according to the following reaction (Equation (1)) [11,12]:

CH_4_ ⟶ C_(solid)_ + 2H_2_ ⟶ ΔH_298 K_ = 74.8 kJ mol^−1^ (1)

High temperatures promote the cleavage of the strong C–H bond in methane, whereas suitable catalysts can substantially reduce the activation energy required for the reaction. As a result of CH_4_ decomposition, the products consist solely of high-purity hydrogen—typically mixed with unreacted methane—and solid elemental carbon. Hydrogen can be separated from unreacted methane using established gas separation technologies, such as pressure swing adsorption (PSA) and membrane-based processes [13,14]. CDM process produces no CO or CO_2_ by-products, thereby eliminating the requirement for any downstream CO_2_ removal steps. Various forms of carbon, including carbon nanotubes (CNTs), carbon nanofibers (CNFs), carbon nano-onions (CNOs), graphitic carbon, or amorphous carbon, can be produced using different reaction conditions and catalyst systems. The solid carbon produced during CDM is not simply a waste byproduct, but a valuable material with potential industrial applications. The solid carbon by-product can be utilized in a wide range of industrial applications, including materials science, energy storage, electronics, and catalysis. Therefore, CDM can be regarded as a dual-benefit process that enables both hydrogen production and carbon material synthesis [15].

Qian et al. [16] reviewed various forms of carbon: CNTs can be used in electrode materials, catalysts, electronic devices, and fuel cells; CNFs have applications in textiles and aerospace technologies; and carbon nano-onions (CNOs) are utilized in lubricants, supercapacitors, and adsorbents for wastewater pollutant removal. Furthermore, CNOs have demonstrated potential for wastewater treatment applications, including the removal of humic acids and micropollutants, with removal efficiencies of up to 98% [17], and for supercapacitors due to their high specific capacity and strong cycling stability [18]. However, a major challenge is the rapid deactivation of catalysts caused by the encapsulation of active metal particles by carbon deposits. Consequently, considerable research efforts have been devoted to improving catalyst performance. Thus, the high cost and limited life-time of catalysts remain major barriers to the large-scale application of the CDM process, which is currently restricted primarily to laboratory-scale studies. This review discusses the fundamentals of methane decomposition—focusing on the design of catalysts, especially Ni-, Fe, and Co-based catalysts; factors affecting methane decomposition, with a special focus on catalyst decomposition and regeneration; and involved reaction mechanisms—followed by the conclusions and outlook.

## 2. Reaction (Deactivation/Regeneration) Mechanism

Initially, methane undergoes molecular chemisorption on the active sites (X), followed by C–H bond dissociation to generate C–X and H–X intermediates (Equations (2)–(7)). In the presence of metal particles, the carbon atom of methane interacts with coordinatively unsaturated metal sites, leading to the formation of M–C σ bonds. The strength of this interaction is strongly influenced by the nature of oxide support. Subsequently, the four C–H bonds of chemisorbed methane are progressively cleaved, resulting in the formation of H_2_ and solid carbon. In this mechanism, the initial activation of methane is widely regarded as the rate-determining step [12,16,19].

CH4 + X ↔ CH4–X(2)

CH_4_–X + X ↔ CH_3_–X + H–X (3)

CH_3_–X + X ↔ CH_2_–X + H–X (4)

CH_2_–X + X ↔ CH–X + H–X (5)

2H–X ↔ H_2_ + 2X (6)

C–X ↔ C + X (7)

**Figure 1:**
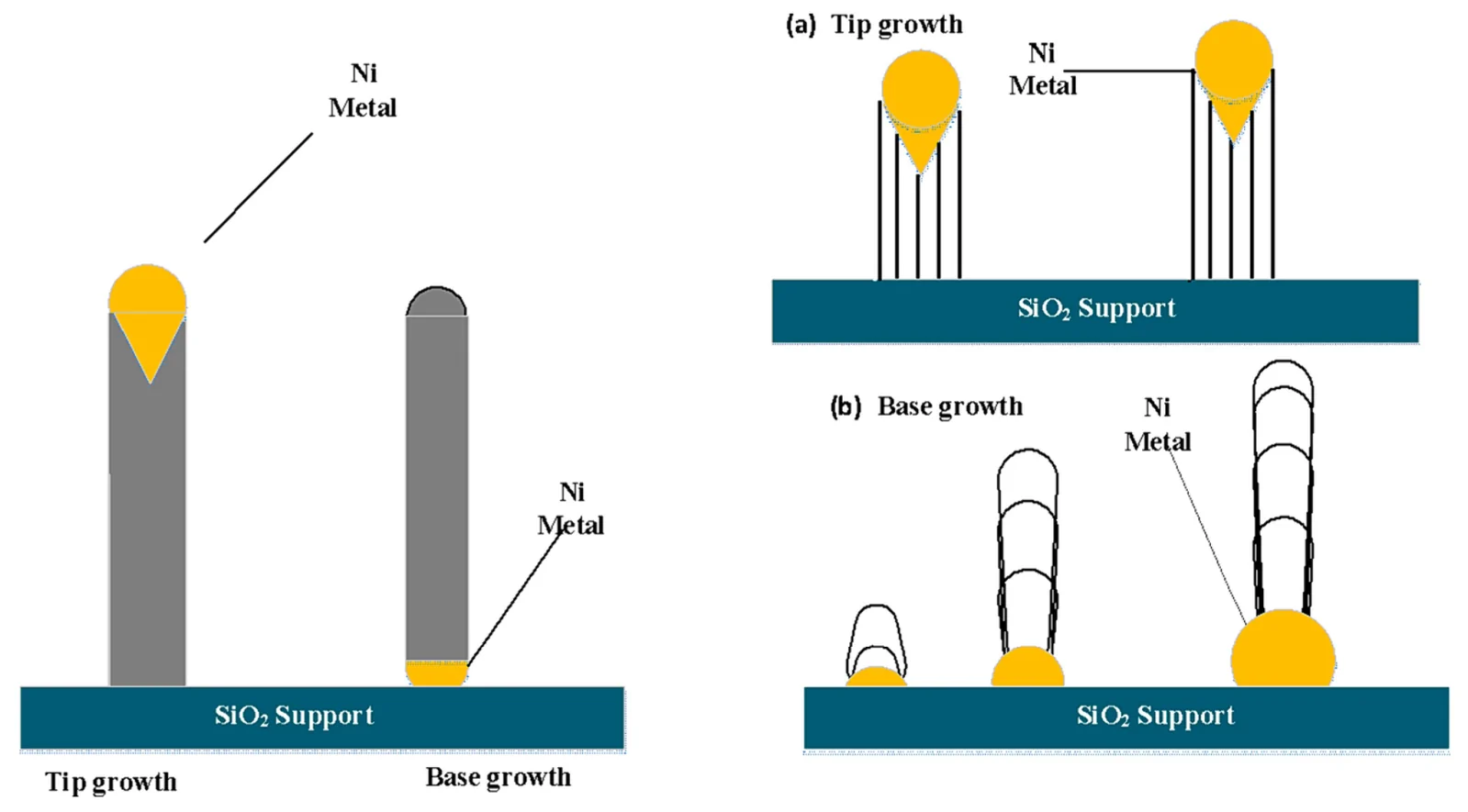
Two growth model diagrams for CNFs: (**a**) tip-growth mechanism and (**b**) base-growth mechanism. Reprinted from Ref. [26] with permission. Copyright 2024, Elsevier.

After methane activation and dissociation, the generated hydrogen is released as a gaseous product, while the remaining carbon atoms accumulate on the catalyst surface, forming encapsulating carbon layers driven by the carbon concentration gradient. Once the carbon concentration reaches saturation, carbon precipitation occurs at the rear surface of the catalyst particles. Carbon nanomaterials subsequently continue to grow on the trailing surface of the catalyst particles through the formation and growth of carbon nuclei. The physicochemical properties of the catalyst, including its composition, structure, and activity, strongly influence the nucleation, growth behavior, and resulting morphology of carbon species formed on the catalyst surface. Two main mechanisms have been widely proposed for methane decomposition: the dissociative adsorption mechanism and the carbide mechanism (e.g., [20,21,22]). In the dissociative adsorption mechanism, methane undergoes direct cleavage on the metal surface, whereas the carbide mechanism involves the initial formation of metal carbide intermediates followed by their subsequent decomposition. The catalyst life-time is strongly governed by the dynamic balance between carbon generation and removal processes, which determines the extent of carbon accumulation and catalyst deactivation. Catalyst deactivation is induced when carbon accumulation on the catalyst surface outpaces carbon diffusion through the metal particles, resulting in the encapsulation of active sites. Under these conditions, excess carbon accumulates on the gas-exposed surface of the metal particles, leading to the formation of graphitic carbon layers, commonly referred to as encapsulating carbon. The formation of encapsulating carbon layers progressively deactivates the catalyst by covering active metal sites and diminishing the accessible surface area [23,24,25]. Catalytic carbon growth follows two main mechanisms: base-growth and tip-growth (Figure 1) [26]. In the base-growth mechanism, the catalyst particle remains anchored to the support while carbon filaments grow outward from the particle. In the tip-growth mechanism, the catalyst particle detaches from the support and remains at the tip of the growing carbon filament. The carbon growth mode is strongly dependent on metal–support interactions; weak metal–support adhesion facilitates catalyst detachment and tip-growth, whereas strong interactions anchor the catalyst particles and favor base growth [27,28,29].

To maintain stable catalytic activity over an extended period in the catalytic decomposition of methane, several process parameters must be carefully controlled. According to the literature (e.g., [30,31,32]), the operating conditions that most significantly influence the catalyst deactivation rate include methane flow rate, reaction temperature, methane partial pressure, and hydrogen partial pressure. Consequently, the large-scale commercialization of CDM relies heavily on the development of effective catalyst regeneration approaches to restore catalytic activity and extend catalyst life-time. To date, three major catalyst regeneration methods have been widely reported in the literature: steam regeneration, air regeneration, and, to a lesser extent, CO_2_ regeneration [33]. Steam regeneration is a widely investigated strategy for recovering deactivated catalysts by removing carbon deposits through steam-assisted gasification, generating CO and H_2_ as products. Compared with air regeneration, this approach offers the advantage of restoring active sites while limiting excessive oxidation of the active metal phase. However, excessive steam exposure or prolonged regeneration may lead to catalyst oxidation, metal sintering, and structural alterations of the support. Therefore, optimizing steam regeneration conditions is crucial to achieve efficient carbon removal while preserving catalyst stability and catalytic performance [12,34]. Air regeneration enables rapid and effective removal of carbon deposits from deactivated catalysts through oxidative combustion, converting deposited carbon into CO_2_. However, the combustion of carbon deposits during regeneration produces large amounts of CO_2_, with emissions nearly comparable to those from the steam methane reforming (SMR) process [35,36,37]. CO_2_ regeneration has emerged as an alternative approach for recovering deactivated catalysts by removing carbon deposits via the reverse Boudouard reaction, where deposited carbon is gasified to produce CO. Compared with air regeneration, CO_2_ treatment provides a milder oxidizing environment, which helps minimize the oxidation of metallic active species and maintain catalyst structural integrity. However, the relatively sluggish kinetics of CO_2_-mediated carbon gasification often necessitate elevated regeneration temperatures or extended treatment durations to achieve effective carbon removal [38].

The paradox of generating CO_2_ during catalyst regeneration fundamentally undermines the environmental premise of this process. To address this limitation, considerable research has focused on the development of non-destructive catalyst regeneration strategies that avoid carbon oxidation [39]. Among these, physical carbon removal has emerged as a promising approach, particularly for transition-metal catalysts (e.g., Fe-, Ni-, and Co-based systems) that predominantly produce filamentous carbon structures [40]. Owing to the weak interaction between filamentous carbon and active metal particles, carbon nanofibers or nanotubes can be removed through mechanical vibration, fluidization, ultrasonic treatment, or continuous reactor configurations, thereby exposing active sites while preserving catalyst integrity. Unlike conventional oxidative regeneration, these methods retain the carbon as a value-added product and eliminate CO_2_ emissions associated with carbon combustion, offering significant advantages in terms of process sustainability [41]. Nevertheless, their applicability remains highly dependent on the nature of the carbon deposits, catalyst structure, and reactor design. In particular, the efficient removal of encapsulating or amorphous carbon remains a major challenge, limiting the universal implementation of physical regeneration strategies [40].

Chemical looping has recently attracted increasing attention as an alternative strategy for sustainable catalyst regeneration [42]. Unlike conventional oxidative regeneration, this approach employs oxygen carrier materials to supply lattice oxygen indirectly, enabling controlled oxidation of deposited carbon while reducing direct exposure of the catalyst to gaseous oxidants [43]. Such controlled oxygen transfer can mitigate catalyst over-oxidation, suppress thermal degradation, and improve the stability of the active metal phase over repeated regeneration cycles. In addition, the cyclic reduction–oxidation behavior of oxygen carriers offers opportunities for enhanced process integration, particularly with CO_2_ capture and utilization technologies, thereby improving the overall environmental performance of CH_4_ decomposition. Carefully designed chemical looping schemes may also enable partial or continuous catalyst regeneration without completely interrupting CH_4_ decomposition, offering the potential for higher process efficiency and prolonged catalyst life-time. Despite these advantages, further optimization of oxygen carrier materials, regeneration conditions, and reactor configurations is required to achieve stable long-term operation and practical industrial implementation [39].

From a techno-economic perspective, conventional in situ oxidative regeneration remains an attractive option because of its operational simplicity, relatively low capital investment, and technological maturity [39]. However, the associated CO_2_ emissions, progressive catalyst degradation caused by repeated oxidation–reduction cycles, and irreversible loss of valuable solid carbon diminish its long-term environmental and economic viability. In contrast, non-oxidative regeneration strategies, particularly physical carbon separation, offer the dual advantages of eliminating direct CO_2_ emissions during regeneration and preserving carbon products for subsequent valorization. The recovery of carbon nanomaterials as value-added products has the potential to generate additional revenue streams, thereby improving the overall economics of CH_4_ decomposition. These benefits, however, are accompanied by increased process complexity, including the need for advanced reactor configurations, continuous carbon removal systems, and higher capital investment. Consequently, future commercial CH_4_ decomposition technologies are expected to increasingly favor regeneration strategies that simultaneously extend catalyst life-time, maximize carbon valorization, and minimize greenhouse gas emissions, highlighting the importance of integrating catalyst design, reactor engineering, and process optimization to achieve economically competitive and environmentally sustainable H_2_ production [44,45].

## 3. Catalysts for Methane Decomposition

Thermal decomposition of CH_4_ typically requires temperatures above 1200 °C; therefore, catalysts are essential for achieving significant methane conversion under milder operating conditions. The elimination of oxidizing agents, including oxygen and steam, represents a key feature that differentiates CDM from conventional methane conversion technologies. Although the reaction equilibrium favors hydrogen production at elevated temperatures, practical implementation requires efficient catalysts to reduce activation energy barriers and enhance reaction rates. A significant advantage of CDM lies in its production of hydrogen without direct CO_2_ formation, potentially simplifying downstream purification requirements and improving process efficiency. However, catalyst deactivation (as indicated above) caused by carbon deposition remains a critical challenge that must be overcome to achieve stable long-term operation. Active metals, supports, and promoters play crucial roles in methane decomposition, significantly influencing catalytic activity, hydrogen yield, and catalyst stability. Recent research has focused extensively on catalyst optimization strategies aimed at simultaneously improving activity, selectivity, and long-term stability [16,46]. Among unsupported metal catalysts, the intrinsic activity toward methane decomposition varies significantly, following the order: Pd, Cu, W, Fe, Mo < Pt, Re, Ir < Co, Ru, Ni, Rh [47]. Qian et al. [16,46] assessed hydrogen costs for CDM using Ni-, Fe-, and Pd-based catalysts and found that the hydrogen cost follows the decreasing trend: noble metal catalysts (USD 1785.7/mol H_2_) ≪ Ni-based catalysts (USD 0.89/mol H_2_) ≪ Fe-based catalysts (USD 0.009/mol H_2_). Transition metals, particularly those in group VIII of the periodic table (i.e., Ni, Fe, Co), have also been widely employed in the CDM. Under methane decomposition conditions, CH_4_ can promote the reduction of iron oxide, nickel oxide, and cobalt oxide, leading to the formation of metallic Fe, Ni, and Co active phases, respectively. The type of metal, its dispersion, and its interaction with the support all strongly affect catalytic activity and stability. The support (e.g., MgO, Al_2_O_3_, activated carbon, ZSM-5, etc.) plays a role beyond that of a mere carrier [48]. Metal–support interactions can profoundly affect catalytic performance by tuning the geometric structure, electronic properties, and surface characteristics of the active metal phase. Accordingly, achieving high catalytic performance requires the rational optimization of active phase–support interactions through the selection of suitable catalyst components. Generally, studies focusing on CDM can be divided into those focused on hydrogen production (e.g., [49,50]) and those targeting the production of diverse carbon materials (e.g., [51,52,53]). Yang et al. [54] presented the chronological development of catalysts and experimental technologies for CDM studies. In our review, we focus on the catalyst composition for hydrogen production and carbon species developments over Ni-, Fe-, and Co-based catalysts with respect to factors that affect the reaction, such as temperature, gas flow rate and pressure, reactor type and operating mode.

### 3.1. Ni-Based Catalysts

#### 3.1.1. Metallic and Supported Catalysts

Tokunaga et al. [55] reported that when Ni is used as the active component, it exhibits higher catalytic efficiency than Fe, along with improved stability and better resistance to coking. Comparative studies have demonstrated that the methane decomposition activity of iron-group metals decreases in the following sequence: Ni > Co > Fe [56,57]. Because CDM is an endothermic reaction, higher temperatures are required to achieve high methane conversion. However, due to the limited thermal stability of Ni catalysts at temperatures above 600 °C, modifying them with promoters or suitable support materials is an effective strategy to improve both catalytic activity and stability [58,59]. Nickel-based catalysts generally show superior CDM activity, with reported activation energies ranging from 26.8 to 88.2 kJ mol^−1^, compared with cobalt- (56–88 kJ mol^−1^) and iron-based catalysts (27–160 kJ mol^−1^), respectively [60,61,62,63,64,65,66,67]; however, their practical application is limited by rapid deactivation resulting from the formation of encapsulating carbon layers that block active metal sites. Optimal operation has been observed within a narrow temperature range of 500–550 °C, whereas temperatures above 600 °C led to rapid catalyst deactivation. Furthermore, they can catalyze the formation of carbon filaments. The formation of a stable NiC*_x_* carbide intermediate has been proposed by some researchers as a key species involved in methane decomposition (e.g., [68,69,70]). Conversely, other studies have revealed that metallic Ni constitutes the active phase, with graphene formation governed by the migration and diffusion of surface and subsurface carbon species. According to this mechanism, monoatomic Ni step edges located at the C–Ni interface are reconstructed by incorporating diffusing carbon species, accompanied by the displacement and migration of Ni atoms toward the leading edge of the nanoparticle. This process ultimately leads to the tip-growth of CNTs (e.g., [71,72,73]). Zhang et al. [74] reported one of the longest CDM life-times, with unsupported Ni-based catalysts remaining active for up to 100 h at 500 °C and a GHSV of 45 L g_cat_^−1^ h^−1^ before deactivation. Despite the extended stability, the catalyst showed relatively low CH_4_ conversion (<10%), corresponding to a H_2_ yield of 80.31 mmol g_Ni_^−1^ h^−1^. Furthermore, the formation of an inseparable Ni-CNT mixture after the reaction poses a significant challenge for practical commercialization. In contrast to unsupported Ni-based catalysts, Ni-supported catalysts generally exhibit much shorter operational life-times in CDM, with most studies reporting reaction times below 5 h. The poor long-term stability of Ni-supported catalysts is generally attributed to non-uniform metal dispersion and the progressive encapsulation of active metal sites by carbon deposits [75,76]. Among the studies on supported Ni catalysts, Venugopal et al. [77] reported a maximum CDM reaction time of approximately 10 h using a 30 wt.% Ni/SiO_2_ catalyst, achieving a H_2_ yield of 126.80 mmol g_Ni_^−1^ h^−1^. The superior stability was attributed to the formation of relatively large Ni particles (21 nm) at high Ni loading, which effectively mitigated catalyst deactivation. Considerable research efforts have been devoted to developing strategies that enhance catalyst durability and prolong stable operation during CDM, including the addition of structural promoters such as La_2_O_3_, SiO_2_, and MgO to suppress carbon encapsulation on active sites, as well as the use of different preparation methods (e.g., impregnation, fusion, and evaporation-induced self-assembly [78,79,80,81]. However, despite these modifications, most catalysts still deactivated within less than 10 h (e.g., [81,82,83,84,85]).

Guevara et al. [86] reported that, during methane cracking, Ni/SiO_2_ catalysts exhibited the greatest catalytic stability and life-time when the metallic Ni particle size was maintained within the optimal range of 60–100 nm. The catalytic performance and durability of CDM catalysts are strongly governed by the characteristics of the support material and the loading of the active metal phase. Takenaka et al. [87] studied the influence of catalyst supports on Ni-catalyzed methane decomposition and found that Ni supported on silica, titania, and graphite showed superior activity and stability, maintaining ca. 4% CH_4_ conversion after 150 min. In contrast, Ni-supported Al_2_O_3_, MgO, and SiO_2_-MgO exhibited low initial activity (<1%) and rapid deactivation within the first 50 min of reaction. For catalysts with similar specific surface areas, weaker metal–support interactions promoted higher methane conversion by enhancing the catalytic activity of the nickel species. Among the catalysts evaluated, silica- and titania-supported systems exhibited superior performance under the investigated conditions, delivering the highest methane conversions and the longest catalyst life-times.

The structure and textural properties of the support, such as porosity, also strongly influence methane conversion. Studies on methane decomposition over Ni-based catalysts supported on silica materials with different specific surface areas and pore-size distributions have demonstrated that both catalytic activity and catalyst life-time are strongly influenced by the pore structure of the support. Silica supports without a well-defined pore structure were found to be the most effective in enhancing both the catalytic activity and durability of Ni-supported catalysts. Furthermore, the support structure influences both the composition of the outlet gas and the morphology of the deposited carbon. For instance, the high oxygen storage capacity and significant lattice oxygen mobility of ceria supports are considered unfavorable because they promote CO formation [30]. Otsuka et al. [88] investigated a series of Ni-supported catalysts for CDM and demonstrated that both catalyst activity and life-time were strongly influenced by the nature of the support material. At 500 °C, hydrogen production followed the decreasing order: Ni/SiO_2_ > Ni/TiO_2_ > Ni/graphite > Ni/ZrO_2_ > Ni/SiO_2_-Al_2_O_3_ > Ni/Al_2_O_3_ > Ni/MgO-SiO_2_ > Ni/MgO, with Ni/SiO_2_ exhibiting the highest H_2_ yield of 1655 mmol H_2_ g_cat_^−1^. The stability of Ni-supported catalysts was reported to decrease in the order: Ni/MgO > Ni/SiO_2_ > Ni/LiAlO_2_ > Ni/ZrO_2_. Ni/MgO demonstrated the highest durability, sustaining more than 30% CH_4_ conversion for 210 min, and could be regenerated in an O_2_ atmosphere without a prior reduction step [89]. Furthermore, Ni–MgO catalysts containing 10–40 wt.% Ni, prepared via a hydrothermal method, exhibited mesoporous structures and the formation of a NiO–MgO solid solution [90].

Gao et al. [91] investigated methane decomposition over Ni/Al_2_O_3_ and Co/Al_2_O_3_ aerogel catalysts with different metal loadings. The experiments were conducted in a fixed-bed reactor at 650 °C and a GHSV of 42,000 h^−1^. During the first 20 min of reaction, Ni/Al_2_O_3_ aerogel catalysts with metal loadings of 50%, 60%, and 70% achieved maximum methane conversions of 73%, 79%, and 69%, respectively. Although some catalyst deactivation occurred over time, methane conversion remained relatively high after 65 min, reaching 66%, 69%, and 64%, respectively. In contrast, Co/Al_2_O_3_ aerogel catalysts with similar metal loadings showed a much faster deactivation. The initial CH_4_ conversions of 63%, 65%, and 60% decreased significantly to 24%, 38%, and 22%, respectively, after 65 min of reaction. These results indicate that the catalytic performance and stability of Ni/Al_2_O_3_ aerogel catalysts are superior to those of Co/Al_2_O_3_ catalysts for CDM.

Nickel phyllosilicate has emerged as a promising catalyst precursor for the generation of highly active and stable metallic Ni sites [85,92]. Interestingly, the nickel particles remained strongly anchored to the support, promoting a base-growth mechanism rather than the conventional tip-growth mechanism commonly observed for state-of-the-art catalysts [92]. The nickel nanoparticles (NiNPs) also exhibit exceptional resistance to sintering, maintaining their structural integrity under harsh reaction conditions at temperatures up to 750 °C. The reduction of Ni–O bonds, particularly those associated with silicon-bonded oxygen species (Si–O–Ni, commonly termed apical oxygen), requires elevated reduction temperatures owing to the strong Ni–O–Si interactions within the silicate framework. Following activation, the highly dispersed, nanoscale nickel particles efficiently catalyze methane decomposition, yielding carbon nanotubes and H_2_. Unlike previous studies, in situ XRD analysis confirmed that no bulk nickel carbide phase was formed during the reaction.

**Figure 2:**
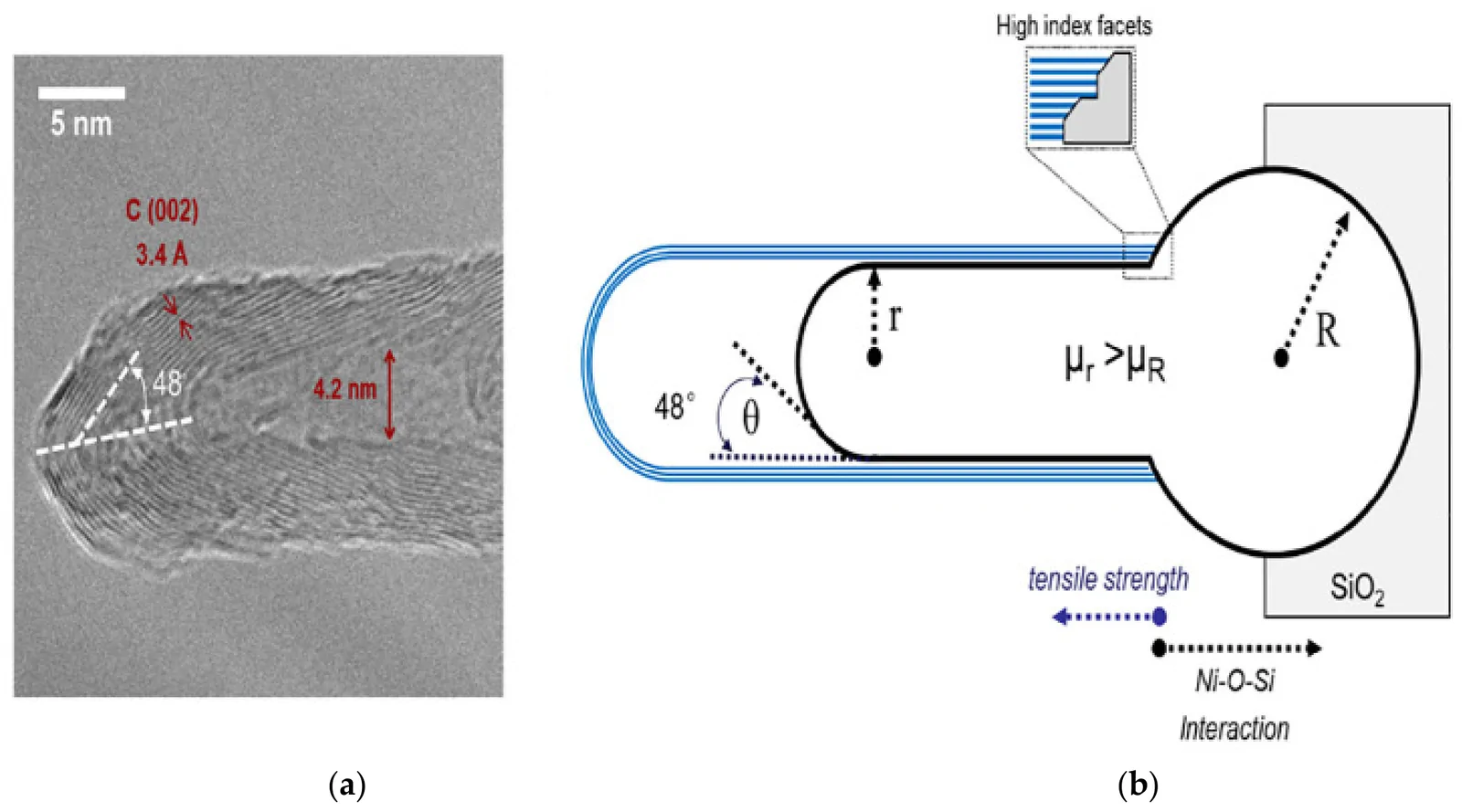
(**a**) HR-TEM of a single carbon nanotube grown in the nickel phyllosilicate showing a base growth mechanism. (**b**) Schematic representation of the dynamics during carbon nanotube growth. Reprinted from Ref. [92] with permission. Copyright 2025, Wiley.

Furthermore, in contrast to conventional nickel-based catalysts, nanoparticles smaller than 5 nm were shown to efficiently activate methane cracking while avoiding carbon encapsulation (Figure 2). This behavior enabled the base-growth of narrow carbon nanotubes with micrometer-scale lengths. Thus, CH_4_ molecules preferentially undergo decomposition at the interface between the active metal sites and the support, as the closed ends of CNTs prevent methane diffusion through the carbon structure [92]. Well-dispersed Ni-SiO_2_ catalysts, obtained by high-temperature reduction of nickel phyllosilicates, show high CDM activity below 500 °C, achieving H_2_ production rates up to 5.3 mol H_2_ g_cat_^−1^ h^−1^ at 25% CH_4_ conversion [85]. Despite expectations that small Ni nanoparticles (<10 nm) rapidly deactivate, their strong performance is attributed to the mobility of Ni-SiO_2_ interfaces in CH_4_ atmospheres, which enables controlled sintering of initially dispersed particles. Furthermore, the ratio of 1:1 to 2:1 nickel phyllosilicates in the precursor, tunable by NH_4_F addition, strongly influences catalyst reducibility and catalytic performance. Awadallah et al. [93] investigated several 50 wt.% Ni-supported catalysts for methane cracking and found that Ni/SiO_2_-Al_2_O_3_ and Ni/SiO_2_-La_2_O_3_ exhibited the highest catalytic activity due to the presence of free or weakly interacting, easily reducible NiO species. In contrast, Ni/SiO_2_–CeO_2_ exhibited superior stability, which was attributed to the enhanced dispersion and facile reducibility of Ni species. The formation of distinct nanocarbon structures was governed by the nature of the support material.

Basalt fiber-supported (Ni/LTA) catalysts were synthesized through surface functionalization, zeolite growth, Ni loading, and activation for low-temperature methane decomposition (500 °C) to produce high-purity H_2_ and multi-wall carbon nanotubes (MWCNTs) [94]. Among the Ni-loading methods investigated, ammonia evaporation (AE) gave enhanced Ni dispersion and catalytic performance compared to deposition precipitation (DP). The 10 wt.% Ni catalyst prepared by AE achieved up to 32% CH_4_ conversion and a H_2_ production rate of 3.1 mol g_Ni_^−1^ h^−1^ over 22 h. When integrated into an LTA/Pd membrane reactor, CH_4_ conversion increased to 45% and reaction time extended to 27 h due to simultaneous H_2_ removal. Compared with Ni/LTA nanopowder catalysts, the basalt fiber-supported catalysts exhibited comparable H_2_ production performance while demonstrating significantly enhanced stability, with a life-time extended by up to 3.7-fold. The catalysts also maintained stable performance during temperature switching and regeneration cycles.

#### 3.1.2. Carbon-Supported Catalysts

Carbonaceous materials, including activated carbon and carbon nanotubes, have been demonstrated to exhibit intrinsic catalytic activity toward methane decomposition. Among the various supported catalyst systems investigated, nickel dispersed on activated carbon has attracted considerable interest because the catalytically active metallic nickel species can be generated in situ during carbonization. This occurs through the reducing capability of activated carbon, which facilitates the transformation of nickel precursors into metallic Ni without requiring an additional reduction step. In related studies, coal char produced from lignite exhibited limited methane decomposition activity, occurring primarily within its microporous structure. Notably, the activation energy associated with C-H bond cleavage in methane over lignite-derived coal char was reported to be substantially lower than that of the non-catalytic reaction. Specifically, the dissociation energy decreased to approximately 89–105 kJ mol^−1^, representing nearly a fourfold reduction compared with uncatalyzed methane decomposition [95]. Prasad et al. [96] synthesized activated carbon from coconut shells and subsequently impregnated it with nickel species to produce a Ni/activated carbon catalyst. During methane decomposition, the catalyst exhibited an initial decline in CH_4_ conversion over the first two hours of reaction. However, after this initial deactivation period, methane conversion gradually increased as reaction time progressed, indicating the development or regeneration of active catalytic sites during the process.

A 10 wt.% Ni-based catalyst-supported carbon derived from coal liquefaction residue, reported by Zhang et al. [97], demonstrated a steady increase in methane conversion from 13% to nearly 60% during 9 h of time-on-stream at 850 °C. The continuous enhancement in catalytic activity was attributed to the gradual generation and exposure of additional active nickel sites during the reaction process. In addition, alternative thermally stable supports capable of maintaining nickel dispersion under high-temperature reaction conditions were also investigated to improve catalyst durability and resistance to sintering.

#### 3.1.3. Alloy Catalysts

Mo incorporation significantly enhanced the dispersion and stabilization of Ni particles in CeO_2_-supported catalysts for methane decomposition at 700 °C [98]. Bimetallic Ni-Mo catalysts supported on CeO_2_ and CeO_2_-SiO_2_ were synthesized and evaluated, revealing that the addition of SiO_2_ negatively affected catalytic performance due to rapid Ni particle agglomeration caused by weak metal–support interactions. In contrast, the Ni-Mo/CeO_2_ catalyst demonstrated superior catalytic activity and stability, attributed to the formation of a stable Ni-O-Ce solid solution and the presence of NiMoO_4_ species, which enhanced Ni dispersion and prevented sintering. The Ni–Mo/CeO_2_ catalyst achieved a hydrogen yield of 48% after 180 min, substantially higher than the 29% yield obtained over the Ni–Mo/CeO_2_–SiO_2_ catalyst. Both catalysts also produced MWCNTs with different morphologies depending on the catalyst structure and composition. Another notable material is Cu-Ni/Al_2_O_3_, which exhibits better performance than Cu/Al_2_O_3_ due to the formation of Ni-Cu alloys. More Ni-Cu alloy formation exhibited superior activity for methane decomposition, while Gd-doped support promoted the formation of a Ni-Cu alloy with a more homogeneous composition [99]. Since alloy phase and composition strongly influence catalytic performance, the development of uniform, well-dispersed alloy catalysts is highly desirable [100]. The reduced (70 wt.%)Ni-(10 wt.%)Cu-(10 wt.%)Fe/Al_2_O_3_ formed a Ni-Cu-Fe alloy, which minimized metal particle contact and prevented sintering [101]. Addition of Fe to (70 wt.%)Ni-(10 wt.%)Cu/Al_2_O_3_ maintained H_2_ concentration between 71–77% and increased carbon diffusion nearly threefold, resulting in a carbon nanofiber yield of 136 g g_cat_^−1^. Wang et al. [102] also reported that (65 wt.%)Ni-(10 wt.%)Fe-(25 wt.%)SiO_2_ achieved 20% CH_4_ conversion at 550 °C. However, Ni-Fe redox species can act as oxygen carriers, potentially affecting CO*_x_*-free H_2_ production [103]. Ni–Fe/Al_2_O_3_ alloy catalysts derived from Ni_3−x_Fe_x_Al (x = 0–1.2) hydrotalcite-like precursors were investigated for CDM at 600 °C to produce H_2_ and carbon materials [104]. Upon calcination at 500 °C, the nickel–iron–aluminum hydrotalcite precursor decomposed to form a Ni(Fe,Al)O solid solution. Subsequent reduction at 800 °C resulted in the formation of a Ni–Fe alloy with a uniform composition and an average particle size of 9.4–10.3 nm. Ni–Fe/Al_2_O_3_ alloy catalysts exhibited a longer catalytic life-time and higher carbon yield than the Ni_3_Al counterpart, with an optimal alloy composition of *n*(Ni)/*n*(Fe) = 4:1. Carbon nanotubes are formed on all catalysts, and Ni-Fe alloying reduces the average carbon diameter to some extent. The Ni–Fe alloy was less susceptible to sintering than metallic Ni under the reaction atmosphere, which may explain the reduced carbon diameter. The catalyst life-time follows the order: Ni_3_Al (3.5 h) < Ni_1.8_Fe_1.2_Al (3.75 h) < Ni_2.7_Fe_0.3_Al (5.25 h) < Ni_2_._1_Fe_0_._9_Al (6.25 h) < Ni_2_._4_Fe_0_._6_Al (7 h). Ni_2_._4_Fe_0_._6_Al exhibits the highest carbon yield, approximately twice that of Ni_3_Al.

**Figure 3:**
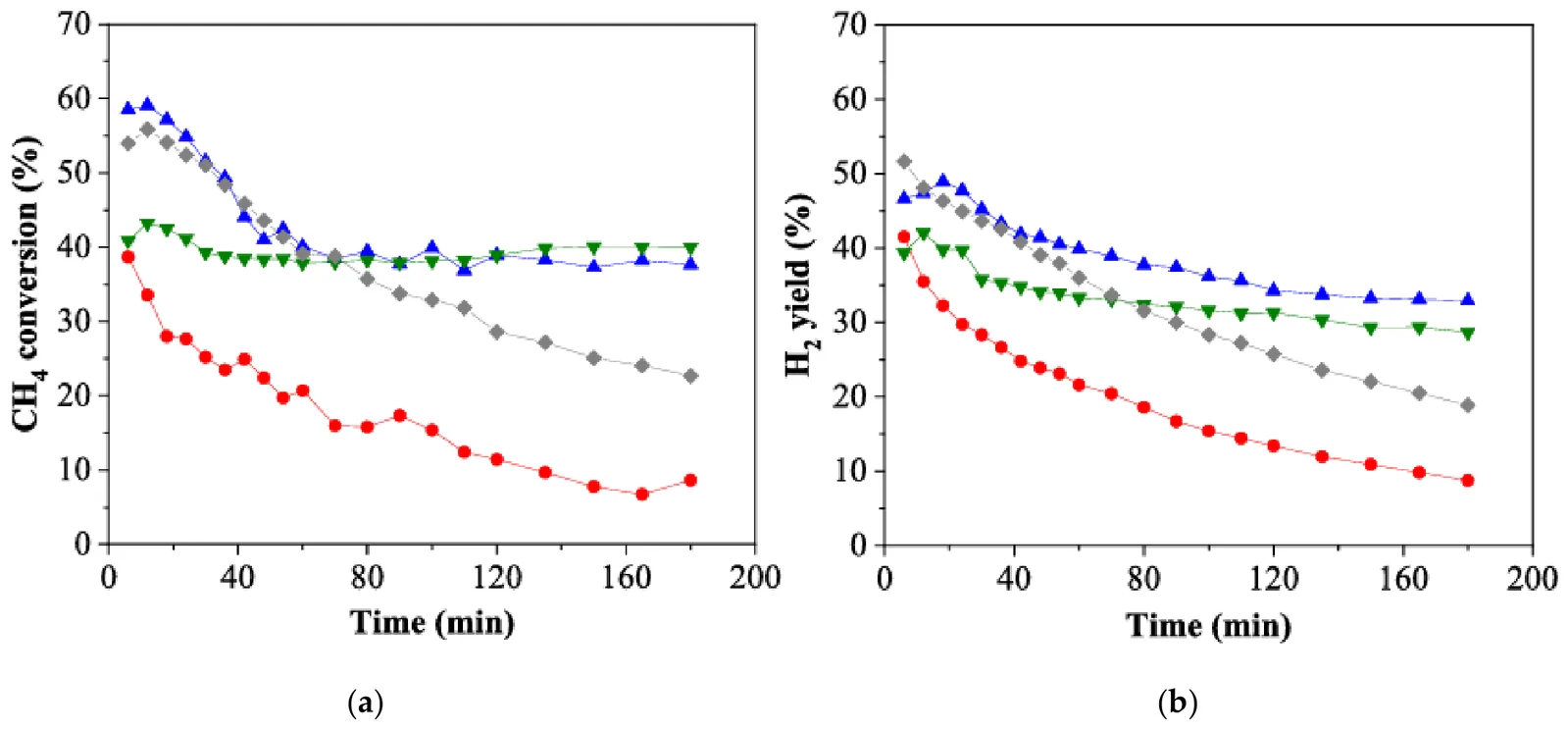
(**a**) CH_4_ conversion and (**b**) H_2_ yield of the NiCu/MS(x) catalyst calcined at different temperatures in methane decomposition at 600 °C. ● (red) NiCu/MS(500), ▲ (blue) NiCu/MS(600), ▼ (green) NiCu/MS(700), and ⧫ (gray) NiCu/MS(800). Reprinted from Ref. [105] with permission. Copyright 2022, American Chemical Society.

Copper-modified nickel-supported mesocellular silica (MS) catalysts, denoted as NiCu/MS(x), were synthesized at various calcination temperatures to enhance both catalytic activity and stability during CH_4_ decomposition at 600 °C [105]. The Ni and Cu active metals were incorporated onto the MS support through a co-impregnation technique, followed by calcination at 500, 600, 700, and 800 °C. Among the prepared catalysts, NiCu/MS(600) exhibited the most outstanding catalytic performance, achieving the highest H_2_ yield of 32.78%, which was approximately 1.47 to 3.87 times greater than the yields obtained from the other NiCu/MS(x) catalysts. In addition to its superior activity, this catalyst also demonstrated enhanced stability throughout the reaction process. The enhanced performance of the NiCu/MS(600) catalyst can be attributed to the beneficial effects of calcination at 600 °C, which promoted improved dispersion of active Ni species, facilitated catalyst reducibility, and strengthened the interaction between NiCu particles and the mesocellular silica support (Figure 3a,b). These characteristics facilitated the formation of fishbone- and platelet-type carbon nanofibers through a tip-growth mechanism during methane decomposition. As a result, the NiCu active sites remained exposed and catalytically active for a longer duration, thereby improving both hydrogen production efficiency and catalyst durability. Moreover, Ni–Cu alloy catalysts have attracted significant attention due to their ability to enhance both catalytic activity and thermal stability under high-temperature reaction conditions. The incorporation of copper into nickel species not only modifies the electronic and structural properties of the catalyst but also enhances resistance to sintering and metal agglomeration during methane decomposition. In addition, the formation of Ni-Cu alloys facilitates carbon diffusion through the metallic lattice owing to the increased lattice spacing and improved interfacial contact within the alloy structure. This characteristic accelerates carbon formation and supports the growth of carbon nanostructures during the reaction process [53,106,107]. Nevertheless, despite these beneficial effects, NiCu catalysts still undergo gradual deactivation during methane decomposition. Catalyst deactivation is primarily associated with excessive carbon accumulation, blockage of active sites, structural changes in the metallic particles, and the eventual encapsulation of catalyst surfaces by inactive carbon species. Consequently, maintaining long-term catalytic stability remains a significant challenge for NiCu-based catalysts in methane decomposition reactions. Ball milling and washcoating were effective methods for preparing gauze catalysts [108]. The NiFe/Al_2_O_3_/Fe-frame catalyst showed high methane decomposition activity, low pressure build-up, and good stability. An initial H_2_ yield of 53% and a low deactivation rate of 3.2% after 500 min at 600 °C were achieved with five or more washcoating cycles without calcination. Catalytic performance was governed by the interplay among active component loading, mass transport characteristics, and the accessibility of active species located on the leeward side of the gauze.

#### 3.1.4. Chemical Looping-Based Syngas Production

The dry reforming of methane (CL-DRM/DC-DRM), involving methane decomposition (CH_4_ ⟶ 2H_2_ + C) followed by the reverse Boudouard reaction (CO_2_ + C ⟶ 2 CO), was also successfully demonstrated over Ni-containing catalysts such as Ni/ZrO_2_ [109] or NiCo/MgAl_2_O_4_ [110]. The NiCo/MgAl_2_O_4_ catalyst exhibited outstanding catalytic activity and long-term stability over more than 20 consecutive cycles of chemical looping dry reforming of methane (CL-DRM). Ordered mesoporous MgAl_2_O_4_ supports were synthesized via an evaporation-induced self-assembly (EISA) method, after which Ni-Co bimetallic oxide nanoparticles were uniformly impregnated onto the support surface. The highly dispersed Ni-Co active phases promoted efficient methane decomposition and subsequent CO_2_ activation, enabling the separate generation of H_2_ and CO products. Metallic Ni nanoparticles served as the primary active sites for CH_4_ decomposition, whereas the oxophilic Co promoter substantially enhanced CO_2_ activation and facilitated coke gasification via the reverse Boudouard reaction. An optimal *n*(Ni)/*n*(Co) ratio of approximately 6, corresponding to the NiCo(2.0)/MgAl_2_O_4_ catalyst with a 12/2 metal composition, provided the best performance due to the abundance of surface oxygen vacancy sites. These oxygen vacancies enhanced carbon conversion and catalyst stability, enabling efficient syngas production with a c(H_2_)/c(CO) ratio maintained at approximately 0.9 over 20 consecutive reaction cycles [110]. Ni-Fe redox catalysts can perform as oxygen carriers for the selective conversion of CH_4_ to syngas through chemical looping steam reforming (CL-SRM) [103]. In this process, CH_4_ is initially decomposed into carbon and H_2_ during the reduction step, followed by steam gasification of the deposited carbon to generate syngas (C + H_2_O ⟶ CO + H_2_O), thereby significantly enhancing CO selectivity. The synergistic contributions of Ni species to CH_4_ activation and Fe species to water splitting, together with the formation of a NiFe alloy in the reduced catalyst, facilitated efficient catalytic methane decomposition. As a result, an equimolar Ni-Fe catalyst achieved CH_4_ conversion of approximately 98% and CO selectivity up to 93% at 900 °C, with CO and H_2_ productivities of 9.6 and 29.0 mol kg_cat_^−1^, respectively. CH_4_–CO_2_ chemical looping with nanostructured Ni-Fe materials enables separate H_2_ and CO production. Small amounts of Ni (0–5 wt.%) increase surface area, reduce oxide crystallite size, and improve redox performance [111]. Ni promoted CH_4_ decomposition for H_2_ generation and enhanced CO_2_ activation for CO formation and carbon removal. Catalyst deactivation was mainly due to carbon deposition, while Ni facilitated carbon regeneration during the CO_2_ re-oxidation cycle.

#### 3.1.5. Summary

Overall, nickel-based catalysts represent some of the most extensively investigated materials for catalytic methane decomposition, owing to their high catalytic activity, cost-effectiveness, and strong capability to activate the C–H bonds of methane. The catalytic performance of Ni-based catalysts is strongly governed by the nature of the support material, Ni dispersion, particle size, and the formation of bimetallic alloys with secondary metals such as Cu or Fe. Supports such as activated carbon, Al_2_O_3_, and SiO_2_ can improve catalyst stability and methane conversion, while bimetallic Ni-based catalysts often show enhanced resistance to sintering and carbon deposition. Ni-Cu and Ni-Fe alloys have been reported to improve hydrogen yield, carbon diffusion, and catalyst durability during long-term operation. In addition, structured catalysts prepared by washcoating or ball milling methods have demonstrated high methane conversion and low deactivation rates. Despite their advantages, Ni-based catalysts remain susceptible to deactivation arising from carbon accumulation and metal sintering under high-temperature reaction conditions.

### 3.2. Fe-Based Catalysts

Abundant and inexpensive Fe-based catalysts can be employed to produce CO_2_-free H_2_ through CH_4_ decomposition. Due to its abundance and low cost, iron is an attractive catalytic component, with the additional advantage that any contamination of carbon products is inherently less toxic than that from Ni or Co. Unlike Ni-based catalysts, Fe-containing catalysts can enable catalytic methane conversion at 700–1000 °C, exhibit strong resistance to deactivation and are widely used for the production of filamentous carbon [40]. Compared with H_2_ reduction, the use of CH_4_ feed for the in situ reduction of Fe-oxide catalysts is cheaper and more practical [112]. This activation method avoids additional H_2_ input and simplifies the CDM process [46]. Choudhary et al. [113] stated that high CH_4_ conversion at elevated temperatures is only achievable when the methane concentration in the feed gas is sufficiently high. Fe oxides can be fully reduced to Fe^0^ by CH_4_, and catalysts reduced with CH_4_ exhibit higher activity than those reduced with H_2_ under the same conditions [114]. The relative contributions of metallic iron and iron carbides to CDM mechanism remain a subject of ongoing debate, as their formation and stability are strongly influenced by the reaction conditions. Metallic Fe is generally regarded as the primary active phase responsible for CH_4_ dissociation, particularly during the initial stages of the reaction or under conditions that limit carburization. Under more severe carburizing conditions, such as higher reaction temperatures, extended time-on-stream, or increased methane partial pressures, iron carbide phases form and contribute to catalyst performance by facilitating carbon dissolution and diffusion through the metal particles, thereby promoting the growth of filamentous carbon. Besides operating conditions, the dominant catalytic pathway also depends on the specific catalyst formulation, which explains the differing conclusions reported in the literature (e.g., [115,116,117,118,119,120]) regarding the respective roles of metallic Fe and iron carbides in CDM. Furthermore, considering the multiple valence states of iron [121], the active Fe species may undergo dynamic electronic redistribution during CH_4_ decomposition due to interactions with CH_x_ intermediates, carbon species, and the catalyst support [122].

#### 3.2.1. Supported Catalysts

Iron-based catalysts for CDM have generally been synthesized through deposition methods on oxide supports such as Al_2_O_3_, SiO_2_, ZrO_2_, CeO_2_, MgO, TiO_2_, and calcium silicate MCM-41, as well as in unsupported systems (e.g., [47,115,116,123,124,125,126,127,128,129,130]). For instance, CH_4_ conversions of 43% and 58% were achieved over Fe supported on SiO_2_ and Al_2_O_3_, respectively [131,132]. The conversion of methane into hydrogen and filamentous carbon performed over Fe_2_O_3_/Al_2_O_3_ and Fe_2_O_3_/SiO_2_ (7–77 wt.% Fe_2_O_3_) at 800 °C revealed higher activity over alumina-supported iron oxides. The catalytic activity strongly depended on the particle size of the active species. The structure of carbon formed during CH_4_ decomposition was dependent on the catalyst support. During CDM over Fe_2_O_3_/Al_2_O_3_, multiwalled carbon nanotubes and chain-like carbon fibers were formed. In contrast, Fe_2_O_3_/SiO_2_ catalysts produced chain-like carbon fibers together with carbon fibers composed of numerous spherical carbon units. The carbon yield from CH_4_ decomposition over Fe_2_O_3_/Al_2_O_3_ (22.5 g-C/g-Fe) was higher than that over Fe_2_O_3_/SiO_2_ (7.5 g-C/g-Fe). Fe_2_O_3_ crystallites with diameters below approximately 30 nm in the fresh catalysts were rapidly converted to α-Fe and Fe_3_C (cementite) upon exposure to methane at 800 °C (Fe_2_O_3_ ⟶ Fe_3_O_4_ ⟶ FeO ⟶ α-Fe metal and Fe_3_C), whereas larger crystallites were converted into γ-Fe metal saturated with carbon atoms (austenite) [40]. Redox supports such as TiO_2_ and ZrO_2_ are unsuitable for high-temperature catalytic applications because of phase transitions from anatase to rutile in TiO_2_ and from tetragonal to monoclinic phases in ZrO_2_. Fe species supported on TiO_2_ displayed limited catalytic activity and poor stability and were found to be inappropriate for CH_4_ decomposition [133]. On the contrary, Ago et al. [134,135] investigated CH_4_ decomposition over colloidal Fe nanoparticles (FeNPs) supported on MgO. Their study demonstrated that 10 nm FeNPs did not support CNT growth, whereas 4 nm FeNPs promoted the formation of double- and single-walled CNTs, although only in very low yields, as confirmed by TEM analysis. The low catalytic activity of the 10 nm FeNPs was attributed to their larger particle size, which may have limited CH_4_ diffusion under the reaction conditions or reduced the particle reactivity required to initiate C–H bond cleavage. The results of Wirth et al. [136] and Yoshida et al. [117] indicated that the formation of iron carbide is not necessarily required for CNT growth, as the process strongly depends on the specific iron phase present under the reaction conditions.

The Fe loading on Al_2_O_3_ was varied to investigate its effect on catalytic performance. The highest H_2_ yield (82.25%) was achieved at 12% Fe loading, where the catalyst maintained stable activity for 3 h on stream. Increasing the Fe content beyond this level reduced Fe dispersion, thereby decreasing the available surface area of active sites [54]. SEM and TEM analyses revealed that the CNTs possessed an average outer diameter of 60–80 nm and exhibited well-graphitized nanotube structures with an interlayer spacing of 0.34 nm. Fe-based catalysts supported on Al_2_O_3_ revealed slightly higher catalytic activity than MgO- and TiO_2_-supported catalysts when the Fe loading exceeded 35 wt.% [137]. A CH_4_ conversion of 69% at 700 °C was achieved using a 60 wt.% Fe/Al_2_O_3_. In contrast, Fe/MgO catalysts demonstrated the highest initial CH_4_ conversions at Fe loadings under 30 wt.%. In particular, the 15 wt.% Fe/MgO achieved 63% CH_4_ conversion and maintained stability over 1 h of operation. Conversely, Fe/TiO_2_ catalyst was found to be unsuitable for CH_4_ decomposition due to its poor catalytic performance and unstable support, as mentioned above.

**Figure 4:**
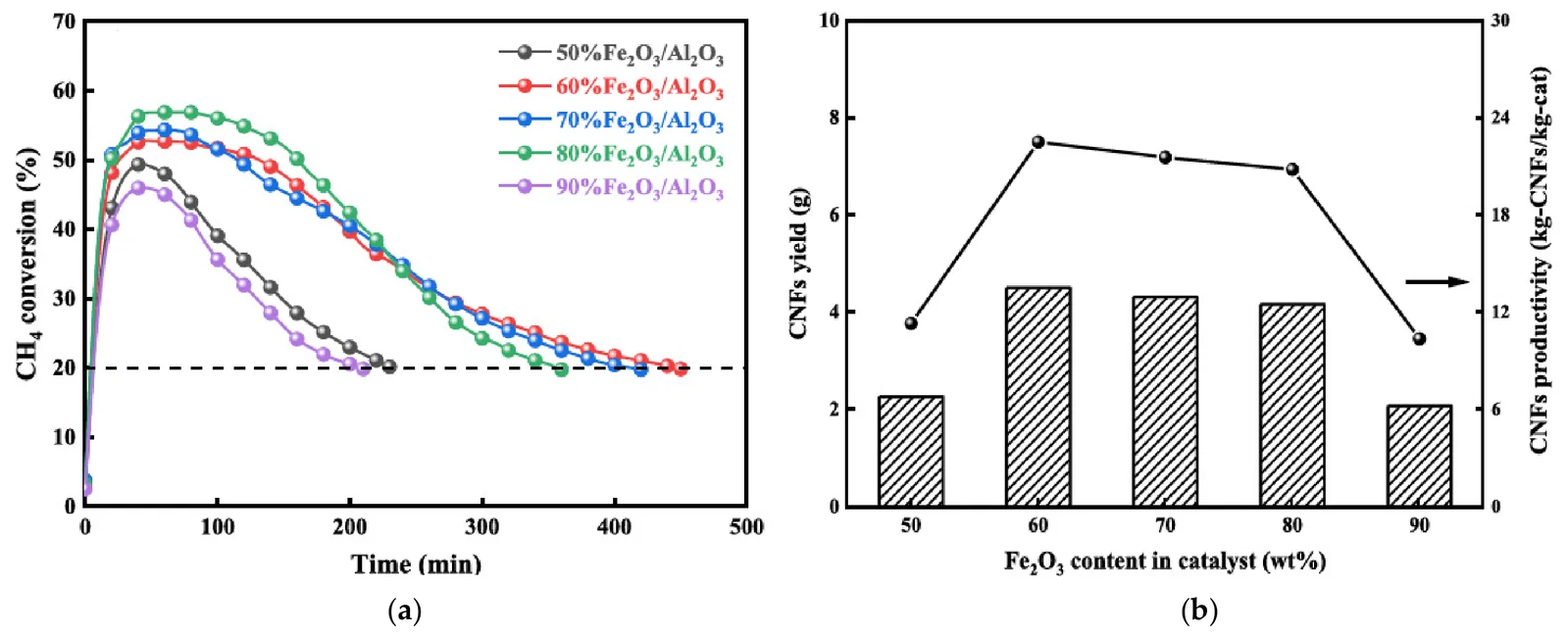
(**a**) Time-dependence of CH_4_ conversion recorded in the CDM tests over five Fe_2_O_3_-Al_2_O_3_ catalysts at 0.1 MPa and 700 °C and a CH_4_ space velocity of 15 000 mL/g^−1^ h^−1^; (**b**) the CNF yields and productivities of the CDM tests of (**a**). Reprinted from Ref. [139] with permission. Copyright 2025, Elsevier.

Without Al_2_O_3_, reduced Fe species underwent severe sintering during the reduction of Fe-based catalysts [138]. Recently, Fe_2_O_3_-Al_2_O_3_ catalysts containing a high Fe_2_O_3_ loading (50–90 wt.%) were prepared via co-precipitation and evaluated for CH_4_ decomposition to produce CNFs with high purity [139]. Catalytic tests conducted at 700 °C demonstrated that Fe_2_O_3_–Al_2_O_3_ catalysts containing 60–80 wt.% Fe_2_O_3_ achieved a maximum CH_4_ conversion of approximately 56% while producing high-purity CNFs (>95%) with a relatively narrow diameter distribution (20–40 nm) and a well-developed graphitic nanostructure (Figure 4a). Electrical conductivity measurements of the obtained CNFs confirmed their good conductive properties, indicating their potential use in anti-static CNF-reinforced plastic composites. The results showed that the selected catalyst achieved a maximum CH_4_ conversion of 57% and a CNF productivity of 21.0 kg_CNFs_ kg_cat_^−1^ in 6 h at 700 °C, while at 720 °C, it reached a higher CH_4_ conversion of ca 64% but a lower productivity of 14.7 kg_CNFs_ kg_cat_^−1^ in 4.2 h (Figure 4b). The catalytic performance of the 80 wt.% Fe_2_O_3_–Al_2_O_3_ catalyst was evaluated at different reaction temperatures and space velocities. The results showed that increasing the reaction temperature enhanced the maximum CH_4_ conversion but decreased the CNF productivity per unit catalyst mass. At each reaction temperature, CNF productivity could be optimized by lowering the CH_4_ space velocity through either reducing the feed rate or increasing the catalyst loading. Therefore, temperature and CH_4_ space velocity were identified as the key operating parameters influencing CNF productivity over the Fe_2_O_3_-Al_2_O_3_ catalyst.

Additionally, ceria-supported iron catalysts (Fe–CeO_2_) were investigated for their catalytic performance in CDM [140]. A catalyst composed of 60 wt.% Fe_2_O_3_ and 40 wt.% CeO_2_ exhibited optimal catalytic activity and the highest exposed iron surface area. The high dispersion of iron species facilitated the retention of active surface area throughout the reaction. An increase in reaction temperature from 600 to 650 °C resulted in enhanced CH_4_ conversion. Trace CO formation during the reaction, caused by mobile lattice oxygen oxidizing carbon species, helped reduce carbon deposition and maintain catalyst activity. The catalyst also formed filamentous carbon, which prolonged catalyst life.

**Figure 5:**
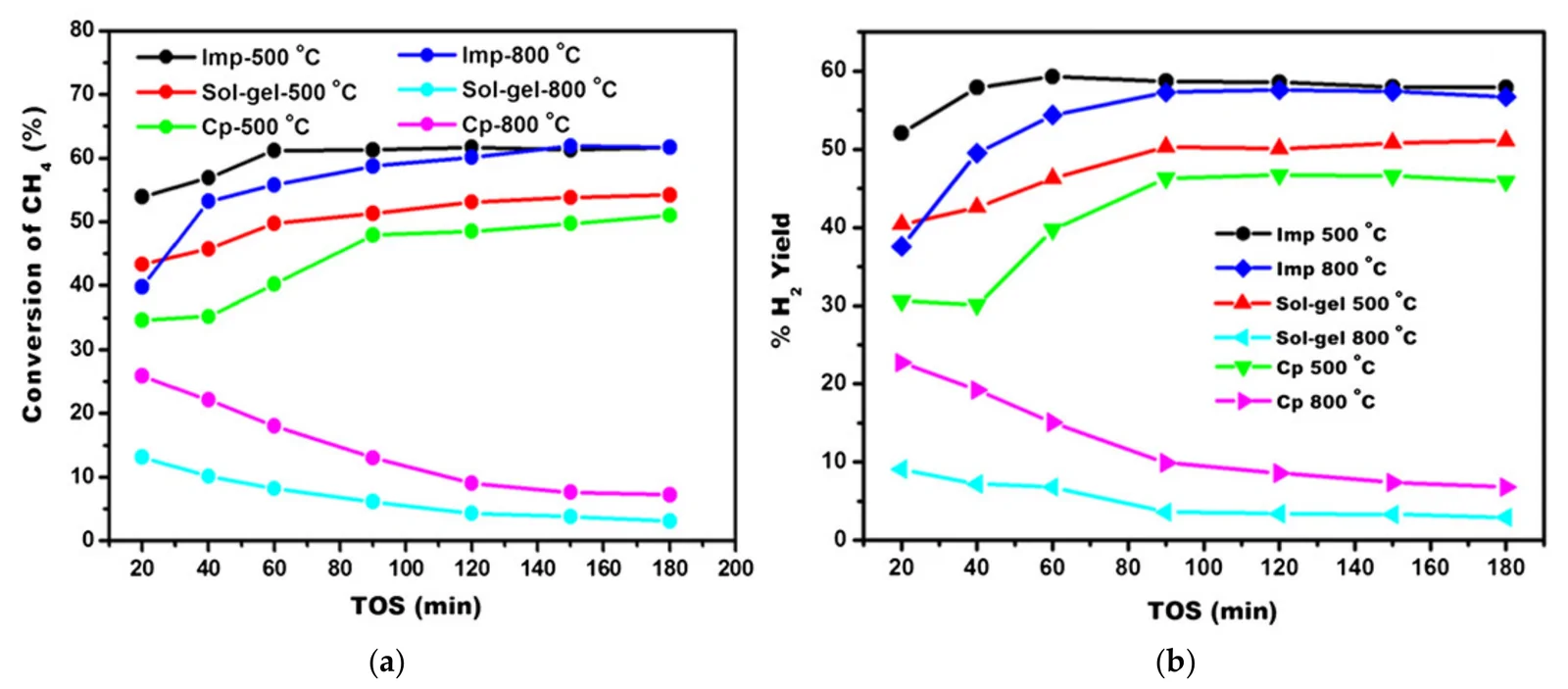
(**a**) Catalyst activity results of 20% Fe/Al_2_O_3_ catalyst for CH_4_ decomposition reaction, (**b**) catalyst activity results of 20% Fe/Al_2_O_3_ catalyst for CH_4_ decomposition reaction in terms of H_2_ yield ((*c*(CH_4_)/*c*(N_2_) = 1.5/1, total flow rate—25 mL min^−1^, space velocity—5000 mL h^−1^ g_cat_^−1^). Reprinted from Ref. [118] with permission. Copyright 2016, King Saud University.

The effects of the preparation procedure and calcination conditions have been broadly studied. Al-Fatesh et al. [118] investigated the influence of calcination temperature on the catalytic activity of alumina-supported Fe-based catalysts prepared via impregnation, sol–gel, and co-precipitation methods. They found that catalysts calcined at 500 °C exhibited higher catalytic activity regardless of the preparation method (Figure 5a,b). However, the catalyst prepared by the impregnation method maintained high catalytic activity even when calcined at higher temperatures. Ni promotion enhanced the reducibility of Fe/Al_2_O_3_ oxides, resulting in improved catalytic activity. The catalyst exhibited stable performance for up to six hours on stream, as confirmed by the catalytic activity results.

#### 3.2.2. Colloidal Iron Nanoparticles and Alloys

**Figure 6:**
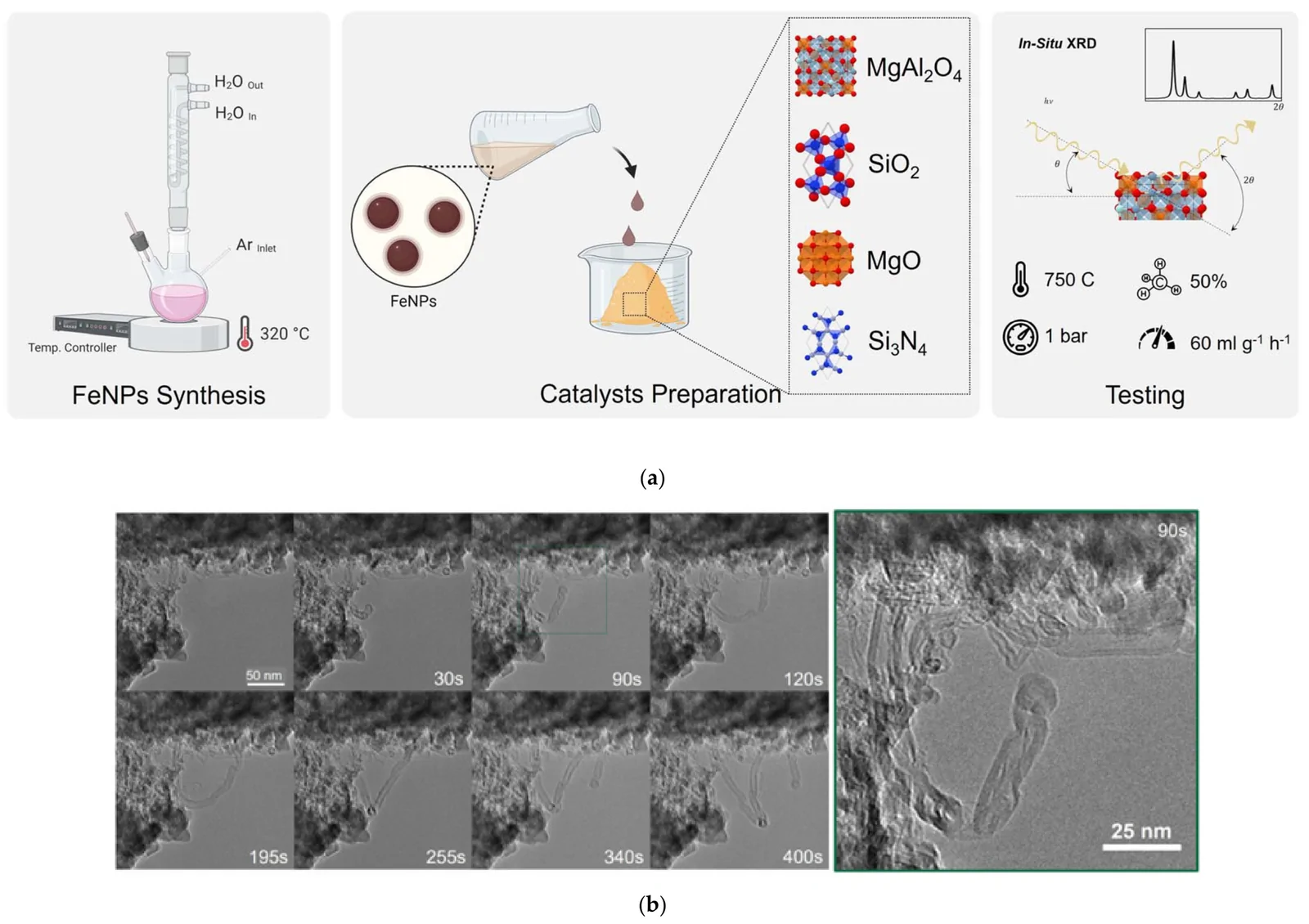
(**a**) Schematic representation of the overall process regarding synthesis, preparation and testing of the supported colloidal nanoparticles. (**b**) Beam-enhanced in situ TEM of FeNPs supported on MgAl_2_O_4_. Time frame indicated in seconds. Reprinted from Ref. [142] with permission. Copyright 2025, Royal Society of Chemistry.

Some studies refer to colloidal iron nanoparticles (FeNPs) deposited on SiO_2_, MgO, Si_3_N_4_, and MgAl_2_O_4_ [119,134,135,141]. Colloidal iron nanoparticles were utilized as pre-formed active sites for the investigation of CH_4_ decomposition [142]. FeNPs supported on SiO_2_, MgO, Si_3_N_4_, and MgAl_2_O_4_ were used to systematically evaluate the influence of support materials on catalytic activity and the characteristics of the resulting carbon products (Figure 6a). Fe/SiO_2_ underwent catalytic deactivation, which was attributed to severe sintering and encapsulation of the active iron species; however, the formation of stable Fe_3_C species was observed under the reaction conditions. Fe/MgO exhibited high resistance to sintering but suffered from serious encapsulation of FeNPs. FeNPs on Si_3_N_4_ maintained their nanoscale size owing to strong metal–support interactions; however, partial reaction of the support with CH_4_ resulted in the formation of C_3_N_3_ species. In contrast, Fe/MgAl_2_O_4_ showed the highest activity and produced abundant CNTs through tip- and base-growth mechanisms. Beam-enhanced in situ TEM confirmed a base-growth mechanism, in which FeNPs remained anchored to the spinel support. MgAl_2_O_4_ was the only support capable of enabling the formation of micrometer-scale CNTs (Figure 6b).

#### 3.2.3. Carbon-Supported Catalysts

The activity of metal-based catalysts (Ni/Al_2_O_3_ and Fe/Al_2_O_3_) and a metal-free catalyst (commercial BP2000 carbon black) for CH_4_ decomposition was investigated in a fluidized-bed reactor between 650–950 °C and GHSV values between 0.9 and 5 L g_cat_^−1^ h^−1^. Among the tested catalysts, Fe_2_O_3_ exhibited the best catalytic performance, achieving the highest CH_4_ conversion (stable for 2 h) of 67%, followed by Ni/Al_2_O_3_ with 48% conversion and BP2000 carbon black with 34% conversion. The BP2000 catalyst showed a slight initial decrease in CH_4_ conversion before reaching stable performance after approximately 60 min. This behavior was attributed to the loss of surface functional groups during the initial stage of the reaction [143]. This series of iron-based catalysts was reduced at different temperatures, revealing that higher reduction temperatures promoted rapid sintering of the iron particles, thereby reducing the efficiency of methane decomposition. Therefore, the optimum reduction temperature for the iron oxide catalysts was determined to be 550 °C [144].

**Figure 7:**
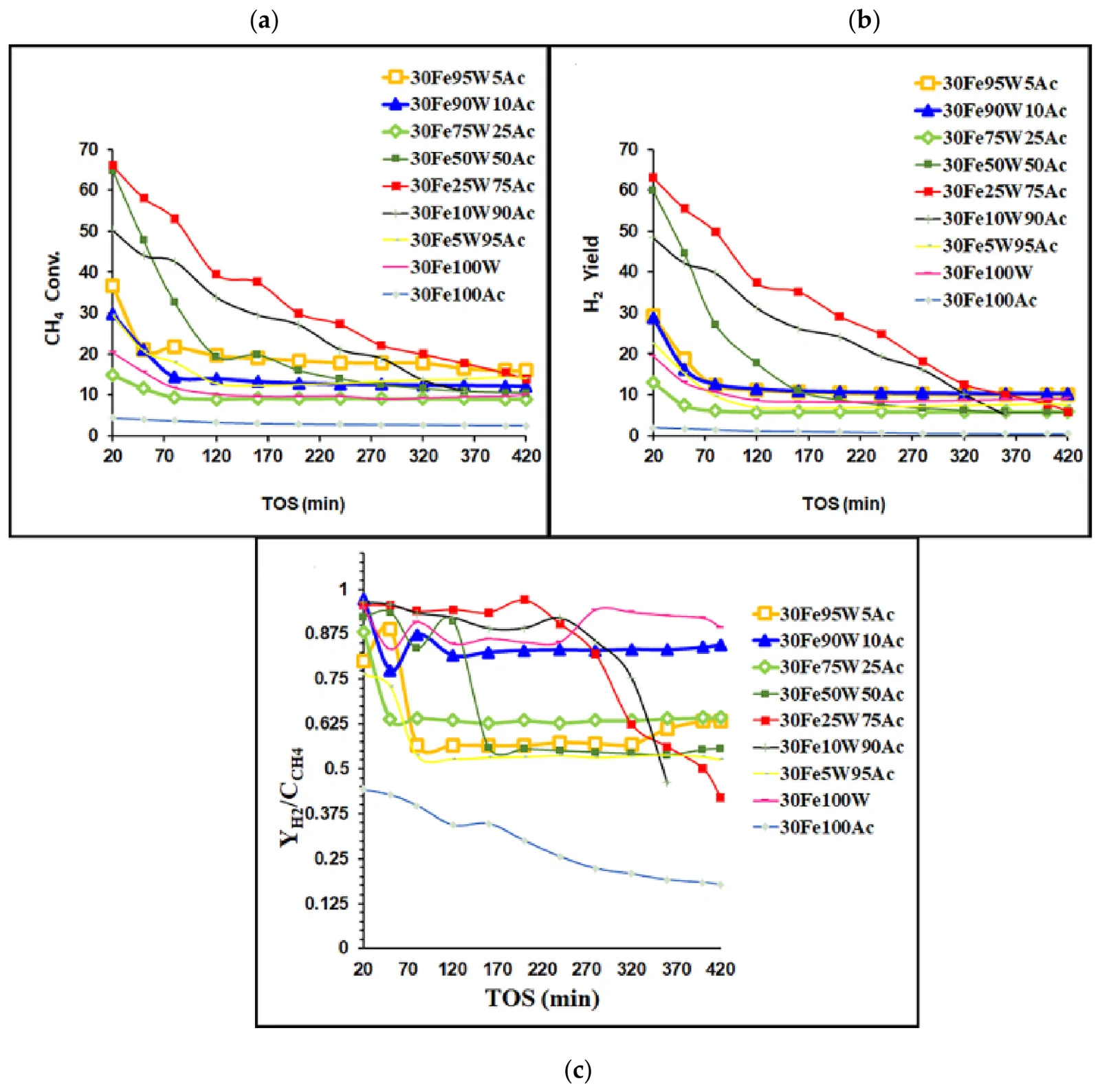
Catalytic activity results of 30Fe*x*W(100–*x*)Ac (*x* = 0–100) catalyst. (**a**) CH_4_ conversion, (**b**) H_2_-yield, (**c**) Y_H2_/C_CH4_ ratio. Reprinted from Ref. [145] with permission. Copyright 2023, Elsevier.

Tungsten oxide-incorporated activated carbon served as an effective support for Fe-based catalysts 30Fe*x*W(100−*x*)Ac (*x* = 0–50) in CH_4_ decomposition, showing higher performance than activated carbon-supported tungsten oxide catalysts (Figure 7a–c) [145]. The enhanced catalytic performance was attributed to the increased surface area and the higher concentration of reducible surface-active species, such as iron oxide, tungsten oxide, and iron tungstate. The 30Fe10W90Ac catalyst exhibited a surface area comparable to 30Fe5W95Ac but showed stronger metal–support interactions, leading to higher catalytic activity. Among the investigated catalysts, 30Fe25W75Ac, consisting of 30 wt.% Fe supported on 25 wt.% WO_3_–75 wt.% activated carbon, demonstrated the highest abundance of reducible active species and superior catalytic performance. It achieved 66% CH_4_ conversion and 63% H_2_ yield, with an initial *c*(H_2_)/*c*(CH_4_) yield ratio above 0.9 that remained above 35% even after 160 min of reaction. In contrast, the highly crystalline 30Fe50W50Ac catalyst exhibited rapid deactivation, which was attributed to the imbalance between carbon formation and carbon diffusion rates. Catalysts with higher W contents (30Fe*x*W(100−*x*)Ac, x = 75, 90, and 95) showed decreased catalytic performance, which was mainly attributed to their lower surface area and limited availability of active catalytic sites.

#### 3.2.4. Doped Catalysts

Promoter metals such as Ni, Co, Cu, Sr, W, La, Pt and Mo have also been shown to improve the activity and stability of the catalysts (e.g., [22,125,129,130,146,147,148]). Fe/ZrO_2_, Fe/La_2_O_3_-ZrO_2_, and Fe/WO_3_-ZrO_2_ (20 wt.% Fe_2_O_3_ supported on metal oxides) were investigated for CH_4_ decomposition over 240 min, and the 20 wt.% Fe/ZrO_2_ catalyst exhibited faster deactivation than the other catalysts [147]. The incorporation of La_2_O_3_ into the catalyst structure enhanced catalyst stability, whereas the use of WO_3_–ZrO_2_ as the support material provided a further improvement in stability. The catalyst containing 20 wt.% Fe/WO_3_–ZrO_2_ maintained stable activity for 240 min, which was attributed to the strong interaction between Fe_2_O_3_ particles and the WO_3_–ZrO_2_ support. The activated carbon support offered the additional advantage of generating catalytically active metallic Fe sites in situ via its intrinsic reducibility [149,150].

**Figure 8:**
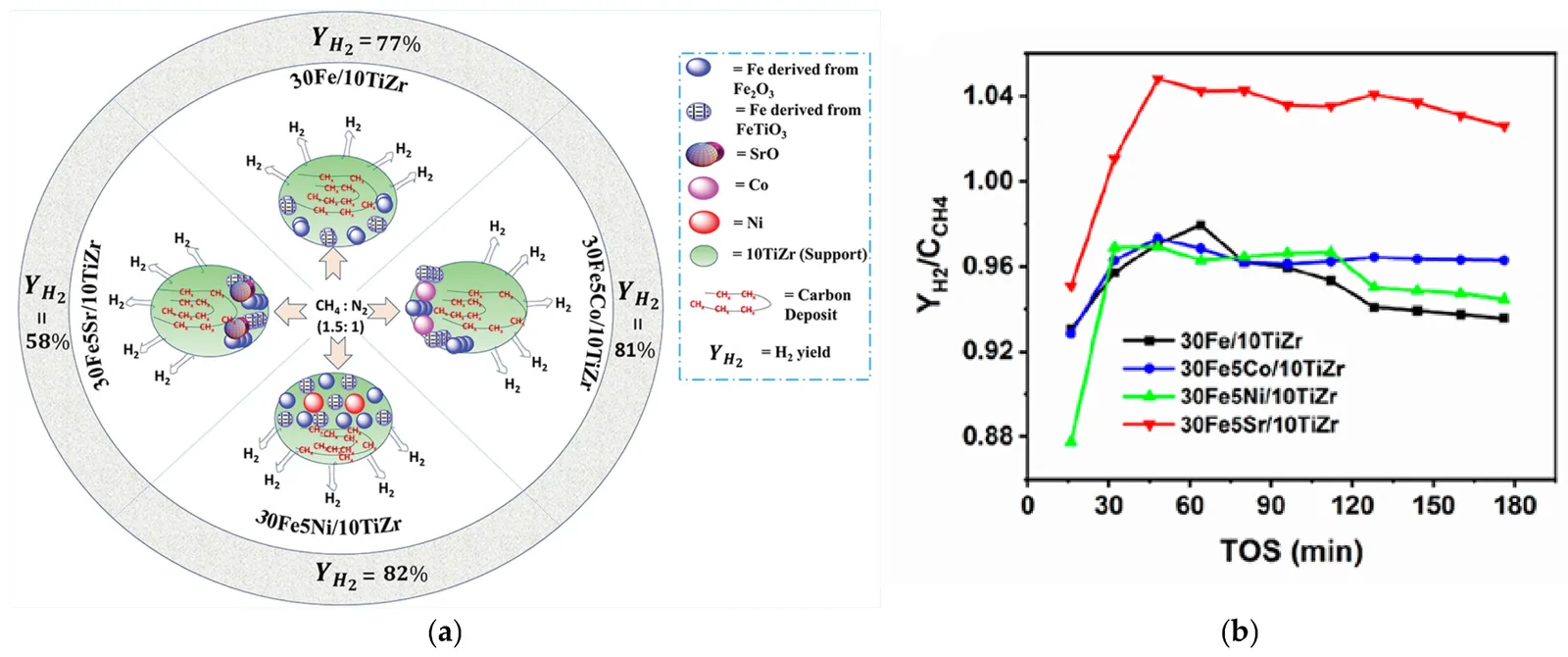
(**a**) H_2_-yield CDM reaction and (**b**) Y_H2_/C_CH4_ vs TOS over Sr, Co, and Ni-modified Fe/10TiZr catalysts. Reprinted from Ref. [122] with permission. Copyright 2024, American Chemical Society.

Pinilla et al. [151] reported an initial CH_4_ conversion of 82% at 850 °C using Fe/Al_2_O_3_ catalysts with a high specific surface area for CDM. In general, the incorporation of metal additives such as Mo, Al, and Zr has been employed to improve the catalytic activity and stability of Fe-based catalysts. It was also reported that the incorporation of 5.1 wt.% Mo into Fe/Al_2_O_3_ modified the catalyst crystallite size and promoted a more homogeneous dispersion of the iron phases. The effect of reaction temperature on Mo-promoted iron-based catalysts revealed that the highest catalytic performance was obtained between 700 and 900 °C. The addition of Al and Zr promoted strong interactions between Fe^0^ and the supports, resulting in catalysts with high stability [35,152]. Titania-modified zirconia dual redox supports (10TiZr) were investigated as support for iron-based catalysts. These supports further stabilized promoter species such as Sr, Co, and Ni at oxygen vacancy sites generated by the Fe^2+^/Fe^3+^ redox pair during CH_4_ decomposition [122]. The introduction of Co or Ni into the 30Fe/10TiZr catalyst (30 wt.% Fe and 10 wt.% TiO_2_) led to the generation of a higher density of reducible species. Notably, the cobalt-promoted 30Fe/10TiZr catalyst exhibited CH_4_ conversion and H_2_ yield values above 80% within 3 h. Additionally, the Ni-promoted 30Fe/10TiZr catalyst achieved a CH_4_ conversion of 87% and a H_2_ yield of 82% after 3 h of continuous operation (Figure 8a,b). Thus, an enhanced reduction profile, in situ generation of active sites by CH_4_ during the reaction, and improved dispersion of active sites are key factors that should be considered for achieving optimal catalytic performance.

Fe-based catalysts supported on metal oxides were prepared via the sol–gel method to investigate the influence of support materials, including yttria (Y_2_O_3_) and Al_2_O_3_, on catalytic activity. The molar ratios of Fe_2_O_3_/Y_2_O_3_/Al_2_O_3_ were 1:0:0, 1:1:0, 1:1:5, 2:1:4, and 3:1:6, respectively [153]. CDM was conducted using Fe-based catalysts, and the catalytic performance was evaluated under a CH_4_/N_2_ flow at both 750 and 800 °C. At 750 °C, the Fe_2_O_3_/Y_2_O_3_ and Fe_2_O_3_/Y_2_O_3_/Al_2_O_3_ catalysts achieved CH_4_ conversions of 29% and 4%, respectively. Alumina addition to the Fe_2_O_3_/Y_2_O_3_ catalyst led to the formation of a garnet-type crystal structure and reduced catalytic activity. The lower methane conversion was attributed primarily to the high dilution of the feed gas and the increased total flow rate. Accordingly, higher CH_4_ concentrations in the feed and lower flow rates are expected to enhance CH_4_ conversion and hydrogen yield. Iron-substituted strontium hexaaluminate materials (SrFe*_x_*Al_12−*x*_O_19_) were synthesized and investigated for their performance in CDM under a concentrated CH_4_ feed (P_CH4_ = 0.9 atm) [154]. The catalysts achieved overall carbon yields of up to 8.08 g_C_ g_cat_^−1^ or 15.68 g_C_ g_Fe_^−1^ at a GHSV of 5 L g^−1^ h^−1^. The high catalytic activity observed for samples with elevated iron content (x ≥ 6) was attributed to the structural collapse of the parent SrFe*_x_*Al_12−*x*_O_19_ phase into α-Fe supported on residual SrAl_2_O_4_ and FeAl_2_O_4_ phases. In situ XRD analysis of SrFe_9_Al_3_O_19_ under CH_4_ atmosphere revealed a sequential phase transformation from α-Fe to γ-Fe and subsequently to Fe_3_C, along with the formation of a small amount of graphite after reaction. These findings suggest that Fe_3_C acts as an active catalytic phase for CDM. Although yttrium and strontium belong to different groups of the periodic table, both have been investigated as promoters in CH_4_ decomposition catalysts due to their ability to modify the structural and surface properties of the active phase.

#### 3.2.5. Waste-Based Catalysts and Natural Ores

The influence of reaction temperature on the performance and product characteristics of rice husk biochar-supported Fe catalysts for CDM was investigated, with particular focus on the role of the biochar support in regulating catalytic activity and carbon product formation [155]. The results demonstrated that catalytic performance improved significantly at 850 °C compared with 750 °C. At 850 °C, CH_4_ conversion, H_2_ yield, and carbon yield reached 24.39%, 739.26 mmol g_Fe_^−1^, and 3.05 g g_Fe_^−1^, respectively, indicating that reaction temperature strongly influenced the activity of the biochar-supported catalyst. Solid-phase characterization demonstrated that the active iron species were present as Fe^0^ before the reaction and underwent transformation into Fe_3_C during the reaction. Owing to the pore structure of the biochar support, the average metal particle size within the mesopores was limited to 21.09 nm. The solid carbon products were predominantly composed of highly graphitized carbon, which was associated with the limited dispersion of active metal species caused by the biochar support. Furthermore, the biochar support facilitated Fe_2_O_3_ reduction, which affected the morphology of the active iron phases and the nature of the carbon products formed. Pore and particle size analyses of the reduced biochar-Fe catalyst further showed that the average mesopore size after pyrolysis-silica solubilization was relatively small (18.98 nm), leading to small surface metal clusters (ca. 21.09 nm), which were unfavorable for CNT growth. Conversely, excessively large pores promoted metal melting and agglomeration at high reduction temperatures, producing large clusters (ca. 740 nm). These large agglomerates became encapsulated by carbon layers, resulting in catalyst deactivation.

An efficient, cost-effective Fe/CaO-based solid waste catalyst was synthesized from red mud (RM) and carbide slag (CS) using a hydrothermal method for CH_4_ decomposition during the catalytic stage and steam gasification of deposited carbon during regeneration [156]. The study also examined the influence of Si, Al, and Ti on cyclic stability. The resulting material was primarily composed of Fe^0^, CaO, CaAl_2_Si_2_O_8_, and CaTiO_3_ phases. Following Na and K removal from red mud, the optimized catalyst composition (25 wt.% red mud and 75 wt.% carbide slag) demonstrated improved cyclic catalytic performance and regeneration stability. In the first cycle, the average CH_4_ conversion reached 85%, while H_2_ production during regeneration was 10.37 mmol g^−1^. After eight cycles, CH_4_ conversion remained relatively stable at 79%. Improved H_2_ production during CDM appeared due to the presence of CaTiO_3_, whose perovskite structure facilitated electron transfer across the crystal surface. Additionally, CaAl_2_Si_2_O_8_ contributed to improved resistance against sintering. Overall, the material showed strong potential for hazardous waste valorization, hydrogen production, and CO_2_ capture.

Cu-, Ce-, Zn-, and Mn-doped Fe/CaO-Ca_12_Al_14_O_33_ catalysts were synthesized by the sol–gel method and evaluated in CDM/regeneration cycles [157]. Among them, the Ce-doped catalyst achieved 90% CH_4_ conversion and 95% H_2_ concentration at the optimal *n*(Ca):*n*(Fe):*n*(Al):*n*(Ce) ratio of 90:10:5:5. Ce doping created oxygen vacancies that enhanced CH_4_ adsorption, CO_2_ capture, electron transfer, and catalyst stability. Steam gasification enabled catalyst regeneration and produced CNT-rich solid carbon with high surface area, demonstrating the catalyst’s strong potential for H_2_ production.

Several recent studies have also highlighted the effectiveness of naturally sourced iron from iron ores or red-mud-derived materials, a by-product of alumina production, as catalytically active materials for CDM (e.g., [120,158,159,160,161]). For example, Balakrishnan et al. [162] used waste red mud from the aluminum industry directly as a catalyst for CDM without requiring a pre-reduction step. The maximum CH_4_ conversion and H_2_ production rate achieved were 19.8% and 3.80 × 10^−5^ mol H_2_ g^−1^ s^−1^, respectively. Zhou et al. [17] investigated iron ore catalysts (with 91.7–96.2 wt.% Fe_2_O_3_, 1.3–2.3 wt.% Al_2_O_3_, 1.2–4.5% wt.% SiO_2_ and 1.3–3.9 wt.% Na_2_O) and reported stable CH_4_ conversion for 5 h at 850 °C and a WHSV of 3.75 L g_cat_^−1^ h^−1^. The results showed that all catalysts exhibited similar activity, while higher Fe content led to improved catalytic performance. Dawkins et al. [163] reduced natural iron ore as an Fe-based catalyst for CDM and identified metallic Fe^0^ as the active phase responsible for CH_4_ decomposition. They also highlighted that the pore structure significantly influences catalyst performance [112,164]. The Australian company Hazer Group developed a multistage fluidized-bed process employing activated iron ore as the catalyst for CH_4_ decomposition (e.g., [165,166,167]). Despite efforts to address technical challenges, the commercial potential of the Hazer Group technology remains limited by the relatively low activity of the iron-based catalyst and the poor quality of the carbon by-products generated during methane decomposition. Other pilot-scale methane pyrolysis demonstration facilities have also been established worldwide, including BASF’s pilot plant in Germany and pilot-scale developments by C-Zero (USA), Ekona Power (Canada), and Hycamite (Finland) [168,169,170,171].

Similarly, low-cost materials, including iron concentrate powder (Fe-IP) obtained by ball milling of iron ore and steelmaking wastes such as fine ash (Fe-FA), coarse ash (Fe-CA), and steel slag (Fe-SS), as well as volcanic mud powder (Fe-VP), have been investigated as catalysts for CDM [172]. The catalysts showed decent CH_4_ conversion at 900 °C for 5 h without H_2_ pre-reduction. Fe_2_O_3_ was gradually reduced by CH_4_ to Fe^0^ (via Fe_3_O_4_ and FeO), which acted as the active phase. Carbon formed on Fe^0^ through Fe_3_C intermediates, producing valuable carbon nanomaterials. For the Fe-IP catalyst, 900 °C and 3 L g_cat_^−1^ h^−1^ were identified as optimal conditions, giving a carbon yield of 4.7 g_C_ g_cat_^−1^. Higher temperature increased CH_4_ conversion, while lower GHSV prolonged reaction time without improving efficiency. The produced CNMs showed good electrochemical performance, with high Li-ion storage capacity (ca. 340 mA g^−1^) and excellent OER activity (1.55 V vs RHE), outperforming commercial IrO_2_.

CH_4_ decomposition was performed over Fe/Al_2_O_3_ catalysts in the absence and presence of O_2_ and CO_2_ [123]. Under an atmosphere containing O_2_ and CO_2_ (*c*(CH_4_)/*c*(O_2_)/*c*(CO_2_) = 80/10/5 vol.-%), deactivation below 825 °C was mainly due to carbon deposition and oxidation of α-Fe and Fe_3_C. Catalyst stability improved with metal addition (ω(M)/ω(Fe) = 1/1 wt.%). CH_4_ conversion over Fe/Al_2_O_3_ decreased from 95% to 79% after 6 h, while Fe/Mg/Al_2_O_3_ maintained 95% conversion, attributed to MgFe_2_O_4_ formation. Stability enhancement followed: Ce, Y > Eu, La > Pr, none > V > Nb.

Fe_2_O_3_/Al_2_O_3_ was employed to systematically investigate the effects of key reduction parameters, including the reducing gas, reduction temperature, heating rate, and heating atmosphere [173]. Under the Fe_2_O_3_/Al_2_O_3_ catalyst composition and experimental conditions investigated in this study, reduction temperatures exceeding 700 °C are likely to be optimal for CH_4_ reduction. The results demonstrate that Fe_2_O_3_/Al_2_O_3_ reduced with CH_4_ exhibits superior catalytic activity compared with the catalyst reduced with H_2_, which is attributed to the formation of active Fe_3_C phases and an increased specific surface area. Increasing the CH_4_ reduction temperature enhanced both the catalytic activity and stability of the catalyst while reducing the required reduction time. In addition, CH_4_ as the heating atmosphere yielded higher catalytic activity than H_2_ or N_2_. The CNTs synthesized under different catalyst reduction conditions exhibited similar morphologies. These findings provide valuable guidance for reactor design and process optimization.

Qian et al. [174] proposed replacing the fixed-bed reactor with a fluidized-bed reactor, which significantly prolonged the operational life-time of the 40 wt.% Fe/Al_2_O_3_ catalyst during CDM by alleviating the pressure drop associated with carbon deposition. The effects of temperature and space velocity were analyzed, showing that higher temperature and lower space velocity improved CH_4_ conversion. At 700–900 °C and 3–6 LN g_cat_^−1^ h^−1^, methane conversion reached 25–40% over 6 h. The process also produced carbon nanofilaments (nanofibers and MWCNTs) similar to those from fixed-bed reactors, but at a higher production scale [175].

#### 3.2.6. Summary

Overall, iron-based catalysts have attracted considerable attention for catalytic CH_4_ decomposition due to their low cost, good carbon tolerance, and ability to produce valuable carbon nanomaterials. Fe-based catalysts are generally less active than Ni-based catalysts for methane activation, but they exhibit greater resistance to deactivation due to carbon deposition. The catalytic performance of Fe-containing catalysts strongly depends on the support material, particle size, and alloy formation with other metals such as Ni or Cu. Ni-Fe catalysts have shown enhanced methane conversion and hydrogen production because Fe species promote carbon diffusion, while Ni facilitates CH_4_ activation. Iron catalysts are also widely investigated in chemical looping processes, where Fe redox cycles can act as oxygen carriers for selective syngas production. However, pure Fe-based catalysts often require higher operating temperatures and suffer from phase transformations and sintering during long-term operation.

### 3.3. Co-Based Catalysts

Cobalt-based catalysts have also been applied for CDM; however, they are less active, raise toxicity concerns, and are more costly compared to nickel-based samples, so studies over Co-based catalysts are fewer. Muradov and Veziroglu [39] summarized preferred operating temperature ranges and the carbon products formed during the CDM process. Cobalt-based catalysts have been investigated for CDM because of their good activity for C-H bond activation and their ability to produce hydrogen and carbon nanomaterials. For CH_4_ activation, Co^3+^ exhibits the highest catalytic activity. Co^2+^ sites exhibit a weaker oxidative effect on CH* intermediates than Co^0^ and Co^3+^ sites. Jin et al. [176] showed that Co(100), CoO(100), and Co_3_O_4_(110) surfaces exhibit distinct CH* oxidation pathways. On Co(100) and CoO(100), CH* primarily reacts with O* to form CHO*, with a significantly greater exothermicity on Co(100). In contrast, on Co_3_O_4_(110), CH* preferentially undergoes reaction with OH* to form CHOH*, followed by its conversion to CO* through the COH* intermediate. This reaction pathway is thermodynamically the most favorable.

Co-containing catalysts generally exhibit lower activity than Ni-based catalysts but often show better resistance to carbon-induced deactivation and metal sintering. The catalytic performance of Co-based catalysts is strongly influenced by the nature of the support material and the dispersion of cobalt species [177,178], and the interaction between cobalt species and the support. Furthermore, the reduction environment of the cobalt precursor plays a critical role in determining both the catalytic activity and the characteristics of the resulting carbon deposits [178]. N_2_ pre-treatment yielded high H_2_ production (ca. 33 mol H_2_ per mol Co after 72 h at 600 °C), which was assigned to smaller Co particle sizes and reduced aggregation, indicating that N_2_ more effectively suppresses sintering than reactive pre-treatment atmospheres. The reduction agent also influenced the carbon structure: H_2_ reduction primarily produced graphitic carbon, whereas CH_4_ reduction favored the formation of thin graphene layers.

#### 3.3.1. Bimetallic Catalysts

Bimetallic catalysts containing Co with Ni, Fe, or Cu have demonstrated improved methane conversion, catalyst stability, and carbon nanofiber formation due to synergistic effects between the metals. Supports such as Al_2_O_3_, MgO, SiO_2_, and carbon materials are commonly used to enhance cobalt dispersion and thermal stability. However, cobalt-based catalysts may still undergo deactivation at high temperatures because of carbon encapsulation and particle agglomeration during prolonged reaction times. According to Zadeh and Smith [179], the initially high rate of CH_4_ decomposition over supported Co catalysts at 450 °C and 101 kPa decreased rapidly but remained active even when the nominal surface coverage of Co by CH_x_ species exceeded unity. To elucidate this behavior, the authors proposed a semi-empirical model involving CH_4_ dissociation on Co active sites followed by the migration of the generated CH_x_ surface species from the metal phase to the support. The migration of CH_x_ species and the subsequent regeneration of active sites were considered essential for explaining the experimentally observed catalyst deactivation profile during CH_4_ decomposition [179].

Zhang et al. [180] evaluated the effects of cobalt dispersion, reaction temperature, and the presence of CO or H_2_ on the kinetic behavior of CH_4_ decomposition over 5–30 wt.% Co/SiO_2_ catalysts. They reported that decreasing Co dispersion from 13% to 5% enhanced the initial CH_4_ decomposition activity and reduced the catalyst deactivation rate. The authors further concluded that filamentous carbon formation contributed more significantly than carbon migration to carbon removal from the metal surface, thereby sustaining catalytic activity during CH_4_ decomposition. Furthermore, the addition of H_2_ or CO suppressed the net rate of carbon deposition and promoted carbon removal through diffusion within the Co particles. Consequently, stable CH_4_ decomposition activity and filamentous carbon formation were achieved over low-loading Co-supported catalysts under appropriate reaction conditions and in the presence of H_2_ or CO. Takenaka et al. [181] reported the performance of Co-based catalysts supported on TiO_2_, MgO, SiO_2_, and Al_2_O_3_. Co/Al_2_O_3_ and Co/MgO exhibited higher activity and stability than Co/TiO_2_ and Co/SiO_2_. They also found that carbon formation during methane decomposition over Co/Al_2_O_3_ is strongly influenced by the reaction temperature. A (20 wt.%)Co/Al_2_O_3_-coated silica fabric catalyst exhibited strong metal–support interactions and maintained 90% CH_4_ conversion for up to 11.6 h at 700 °C [182]. In other studies, the hydrotalcite-derived Co-Al mixed oxide contained Co_3_O_4_ and Co_2_AlO_4_ spinel phases. Reduction in CH_4_, rather than H_2_, produced smaller Co particles and resulted in 75% CH_4_ conversion at 750 °C. In this catalyst, the Co_3_O_4_ phase promoted graphene formation [183,184].

Other studies also concern mixed supports such as (mesoporous) Al_2_O_3_-TiO_2_ (e.g., [185,186]). For example, Gao et al. [185] prepared a series of Al_2_O_3_–TiO_2_-supported Ni- and Co-based monometallic and bimetallic catalysts with different *n*(Ni)/*n*(Co) ratios using the sol–gel method and investigated their catalytic performance for CDM. Among the investigated catalysts, the Al_2_O_3_-supported 9Ni-1Co catalyst showed the best performance, while the corresponding Al_2_O_3_-TiO_2_-supported catalyst (9Ni-1Co/Al_2_O_3_·TiO_2_) exhibited even higher activity and stability. This enhanced performance was attributed to enhanced metal dispersion on the composite support. In addition, TiO_2_ incorporation mitigated catalyst deactivation by facilitating the transfer and removal of carbon nanotubes. Regeneration studies further demonstrated that both catalysts retained high activity after two regeneration cycles. TiO_2_- and ZrO_2_-supported Ni- and Co-based catalysts were investigated for CDM at 700 °C in a fixed-bed reactor [187]. The TiO_2_-supported catalysts exhibited stronger metal–support interactions, whereas the ZrO_2_-supported catalysts showed weaker interactions and more easily reducible active species. Metal–support interactions strongly affected both catalyst activity and carbon growth mechanisms. The ZrO_2_-supported catalysts showed lower deactivation, mainly due to metal particle sintering, while the TiO_2_-supported catalysts experienced higher deactivation caused by both metal sintering and carbon encapsulation of active sites. Carbon nanomaterials exhibited a tip-growth mechanism over ZrO_2_-supported catalysts, which was attributed to the facile detachment of metal particles, whereas a base-growth mechanism occurred over TiO_2_-supported catalysts due to stronger metal–support interactions. Dasireddy and Likozar [66] investigated the formation of carbon nanomaterials during CH_4_ decomposition over Al_2_O_3_-supported Co–Cu, Co–Fe, and Cu–Fe catalysts. They reported the formation of significant quantities of carbon nanotubes on the surfaces of the bimetallic catalysts. Notably, the monometallic Cu/Al_2_O_3_ catalyst showed low but stable methane conversion, which was attributed to the formation of carbon nanotubes on the catalyst surface. These carbon nanotubes likely acted as an additional support for the active metal, thereby improving methane decomposition activity. Furthermore, carbon nanotubes generated over the Cu–Fe/Al_2_O_3_ catalyst exhibited superior crystallinity and a higher degree of graphitization compared with those produced over the other catalysts.

Other bimetallic systems refer to Co-Mo/MgO [188] (the enhanced activity and stability of the catalyst compared with Co/MgO were attributed to the formation of Mo_2_C, the reduced CH_4_ cracking rate, and the improved balance between the CH_4_ cracking rate and the carbon migration rate [188]) or Ni-Co-Al [189] (which was stable with time-on-stream within 500–700 °C). Notably, the catalytic activity of the 25 wt.% Ni–25 wt.% Co/100 wt.% Al catalyst increased significantly with increasing reaction temperature from 500 to 700 °C after 5 h of operation. Furthermore, Ni-Co-Cu-Zn was reported to be even more active and stable up to 80 h than bi- or trimetallic systems (Ni-Co, Ni-Co-Cu) at 700 °C. The addition of Zn promoted the formation of long, thin carbon nanotubes, while catalysts without Zn produced carbon nanotubes with larger diameters [190].

#### 3.3.2. Summary

Cobalt is more often proposed as a promoter/modifier than as a standalone catalyst for methane decomposition. Compared with nickel and iron, cobalt is much more expensive, far less environmentally friendly, and less active than nickel. Similarly, as for nickel and iron, the activity and stability of cobalt-based catalysts depend on metal–support interactions, the crystallite size of the active phase, the addition of promoters and modifiers, and the conditions of methane decomposition—mainly temperature. However, one of the most interesting features of cobalt-based catalysts is the ability to produce predominantly encapsulating carbon. At first glance, this feature may appear to be a major disadvantage because it drastically reduces operating time and fails to produce valuable carbon materials such as carbon nanofibers and nanotubes. Nonetheless, such catalytic materials can be more easily regenerated because the catalytic structure is, to a lesser degree, destroyed by carbon and can be used in production mainly focused on hydrogen. This could be an alternative to the search for catalytic materials, with a mechanism of growth of carbon nanofibers/tubes from the base, although it has some drawbacks.

## 4. Conclusions

CH_4_ decomposition offers a promising approach for sustainable hydrogen production while minimizing greenhouse gas emissions. Significant progress in catalyst development, particularly involving transition-metal and hybrid catalyst systems, has led to notable improvements in catalytic performance. Table 1 summarizes the three main catalyst types concerning optimal temperature, CH_4_ conversion, catalyst life-time, main deactivation and main limitations.

In addition to producing CO*_x_*-free hydrogen, CDM enables the simultaneous generation of valuable CNMs, such as carbon nano-onions, carbon nanofibers, and carbon nanotubes. Despite these advantages, several challenges continue to hinder the large-scale industrial implementation of CDM. The process is highly endothermic and therefore requires substantial energy input. Moreover, catalyst deactivation arising from carbon deposition remains a key bottleneck, compromising catalyst durability and overall process efficiency. Other key challenges include scaling up the process from laboratory to industrial applications and achieving economic competitiveness with established hydrogen production technologies. The high cost and insufficient long-term stability of catalysts remain key barriers limiting the transition of CDM from laboratory-scale studies toward practical applications. In the near term, it is unlikely to replace conventional large-scale hydrogen production methods, primarily because catalyst deactivation occurs through the accumulation of by-product CNMs on the catalyst surface. Nevertheless, CDM is particularly attractive for decentralized, on-site hydrogen generation at small- to medium-scale industrial facilities, such as local hydrogenation stations. Ongoing research efforts are focused on overcoming these limitations through the development of more durable and efficient catalytic systems. Recent advances include plasma-assisted methane decomposition, which enables operation at lower temperatures; molten metal catalysts that provide self-regenerating catalytic surfaces; and emerging photocatalytic and electrochemical approaches, although these technologies are currently in the preliminary stages of development. In addition, hybrid catalyst systems that combine redox and structural functionalities are being actively explored. Machine learning techniques and advanced characterization methods are also increasingly being applied to design next-generation catalysts with optimized properties and enhanced performance. Continued progress in catalyst design, reaction engineering, and process integration will be critical to fully realize the potential of CH_4_ decomposition as a key technology in the transition toward a low-carbon energy future.

## Figures and Tables

**Table 1:** Key similarities and differences between Ni-, Fe-, and Co-based catalysts in CDM.

Catalyst	Temperature /°C	CH_4_ Conversion/%	Catalyst Life-Time	Main Deactivation	Main Limitation
Ni-based	500–700	60–95	Short–medium	Carbon encapsulation of the active phase, Ni-particle sintering, support-pore blocking by carbon	Active-phase detachment from the support by carbon
Fe-based	700–900	50–90	Medium–long	Carbon encapsulation of the active phase, Fe-particle transformation into iron carbide, support-pore blocking by carbon	High operating temperature
Co-based	650–800	70–95	Medium	Carbon encapsulation of the active phase, Co-particle sintering, support-pore blocking by carbon	High cost, toxic

## Data Availability

No new data were created or analyzed in this study. Data sharing is not applicable to this article.
